# A Hybrid One-Way ANOVA Approach for the Robust and Efficient Estimation of Differential Gene Expression with Multiple Patterns

**DOI:** 10.1371/journal.pone.0138810

**Published:** 2015-09-28

**Authors:** Mohammad Manir Hossain Mollah, Rahman Jamal, Norfilza Mohd Mokhtar, Roslan Harun, Md. Nurul Haque Mollah

**Affiliations:** 1 Institut Perubatan Molekul UKM (UMBI), University Kebangsaan Malaysia (UKM), Jalan Ya’acob Latiff, Bandar Tun Razak, Cheras 56000 Kuala Lumpur, Malaysia; 2 Department of Physiology, Faculty of Medicine, Universiti Kebangsaan Malaysia, Kuala Lumpur, Malaysia; 3 Laboratory of Bioinformatics, Department of Statistics, University of Rajshahi, Rajshahi-6205, Bangladesh; University of Lleida, SPAIN

## Abstract

**Background:**

Identifying genes that are differentially expressed (DE) between two or more conditions with multiple patterns of expression is one of the primary objectives of gene expression data analysis. Several statistical approaches, including one-way analysis of variance (ANOVA), are used to identify DE genes. However, most of these methods provide misleading results for two or more conditions with multiple patterns of expression in the presence of outlying genes. In this paper, an attempt is made to develop a hybrid one-way ANOVA approach that unifies the robustness and efficiency of estimation using the minimum *β*-divergence method to overcome some problems that arise in the existing robust methods for both small- and large-sample cases with multiple patterns of expression.

**Results:**

The proposed method relies on a *β*-weight function, which produces values between 0 and 1. The *β*-weight function with *β* = 0.2 is used as a measure of outlier detection. It assigns smaller weights (≥ 0) to outlying expressions and larger weights (≤ 1) to typical expressions. The distribution of the *β*-weights is used to calculate the cut-off point, which is compared to the observed *β*-weight of an expression to determine whether that gene expression is an outlier. This weight function plays a key role in unifying the robustness and efficiency of estimation in one-way ANOVA.

**Conclusion:**

Analyses of simulated gene expression profiles revealed that all eight methods (ANOVA, SAM, LIMMA, EBarrays, eLNN, KW, robust BetaEB and proposed) perform almost identically for *m* = 2 conditions in the absence of outliers. However, the robust BetaEB method and the proposed method exhibited considerably better performance than the other six methods in the presence of outliers. In this case, the BetaEB method exhibited slightly better performance than the proposed method for the small-sample cases, but the the proposed method exhibited much better performance than the BetaEB method for both the small- and large-sample cases in the presence of more than 50% outlying genes. The proposed method also exhibited better performance than the other methods for *m* > 2 conditions with multiple patterns of expression, where the BetaEB was not extended for this condition. Therefore, the proposed approach would be more suitable and reliable on average for the identification of DE genes between two or more conditions with multiple patterns of expression.

## 1 Introduction

Microarray technology has enabled the expression levels of thousands of genes to be investigated simultaneously. However, this technology poses statistical challenges by virtue of the large number of transcripts surveyed with small sample sizes. The identification of transcripts that are differentially expressed (DE) between two or more conditions is a common task that is undertaken to reduce the dimensionality of the transcripts, as important genes belong to the reduced set of DE transcripts. Useful information regarding the regulatory network can be obtained by associating differential expressions with the genotypes of molecular markers [1]. By assigning DE genes to the list of gene sets, it is possible to obtain useful biological interpretations [2, 3]. Furthermore, the number of DE genes that influence a certain phenotype may be large, whereas their relative proportions are typically small; therefore, identifying these DE genes from among the large number of recorded genes is challenging [4–8].

In general, four types of statistical procedures are used to identify DE genes: (i) classical parametric approaches, such as t-test, Ftest (ANOVA) and likelihood ratio test (LRT)-based asymptotic *χ* ^2^-test; (ii) classical nonparametric approaches [8–11]; (iii) empirical Bayes (EB) parametric approaches [5–7, 12, 13]; and (iv) EB nonparametric approaches [14, 15]. In the classical procedures, DE genes are generally detected based on *p*-values (significance levels) that are estimated either via permutation or based on the distribution of a test statistic, whereas in EB procedures, the posterior probability (*pp*) of differential expression is used to identify DE genes. However, most of the aforementioned algorithms are not robust against outliers [12, 16]. Thus, they may produce misleading results in the presence of outlying transcripts or irregular patterns of expression. Several recent studies have reported that the assumption of normality does not hold for some existing microarray datasets [17]. One of the causes for this breakdown of the normality assumption may be related to the presence of outliers in the data. cDNA microarray data are often corrupted by outliers that arise because of the many steps that are involved in the experimental process, from hybridization to image analysis [12, 16].

Some nonparametric approaches, such as KW [9], are somewhat robust against outliers between two or more conditions with multiple patterns of expression in the case of large sample sizes; however, these approaches are sensitive to outliers in the case of small sample sizes. To overcome this problem, the *β*-divergence-based empirical Bayes (BetaEB) approach [16] was developed for the robust identification of DE genes. This approach performs well in the presence of outlying expressions with up to 50% genes for both small- and large-sample cases. The parameters of the BetaEB approach are estimated based on the expressions of all genes. It can tolerate up to 50% outlying genes if the mean vector is initialized by the median vector. In the presence of more than 50% outlying genes, it is difficult to initialize the shifting parameters (mean vector) of this approach with the good part of the dataset. Therefore, the BetaEB approach occasionally produces misleading results. Note that more than 50% outlying genes may also occasionally occur with at least one patient/tissue sample. Moreover, this approach was not extended for the detection of DE genes in the case of more than two conditions/groups with multiple patterns of expression due to the computational complexity. Therefore, in this paper, an attempt is made to develop a hybrid approach that unifies the robustness and efficiency of estimation in one-way ANOVA using the minimum *β*-divergence method [18–21] to overcome all of the aforementioned problems that arise in the BetaEB approach. The advantage of the proposed algorithm compared to BetaEB is that it performs considerably better than the BetaEB approach for both the small- and large-sample cases in the presence of more than 50% outlying genes with 5% outlier samples for each outlying gene. The major advantage of the proposed algorithm is that it performs well in the case of multiple conditions/groups (*m* > 2) for identifying DE genes with multiple patterns of expression, whereas BetaEB was not extended for multiple conditions/groups (*m* > 2) due to the computational complexity. The proposed method introduces a weight function, which plays a key role in its performance. This weight function assigns smaller weights to outlying observations, thereby ensuring the robustness of the inference. Appropriate initialization of the parameters also improves the performance of the proposed method, as is also discussed in this paper.

## 2 Materials and Methods

Let *x*_*jk*_ be the *k*th observed random expression of a gene in the *j*th condition (*j* = 1, 2, …, *m*;*k* = 1, 2, …, *n*_*j*_), which follows the one-way ANOVA model as expressed below:

$$\begin{array}{c} x_{jk}=\mu_{j}+\epsilon_{jk}, \end{array}$$ (1)

where *μ*_*j*_ is the mean of all expressions of a gene in the *j*th condition and *ϵ*_*jk*_ is the random error term that follows $N(0,\sigma_{j}^{2})$. We wish to test the null hypothesis (*H*_0_) : *μ*_1_ = *μ*_2_ = … = *μ*_*m*_ = *μ* against the alternative hypothesis (*H*_1_) : *H*_0_ is not true, assuming that $\sigma_{1}^{2}=\sigma_{2}^{2}=...=\sigma_{m}^{2}=\sigma^{2}$. Thus, the generalized likelihood-ratio test (LRT) criterion yields the following *F*-statistic to test *H*_0_ against *H*_1_:

$$\begin{array}{c} F=\frac{\sum_{j=1}^{m}n_{j}{\left({\hat{\mu }}_{j}-\hat{\mu }\right)}^{2}/\left(m-1\right)}{\left[n_{1}{\hat{\sigma }}_{1}^{2}+n_{2}{\hat{\sigma }}_{2}^{2}+...+n_{m}{\hat{\sigma }}_{m}^{2}\right]/\left(n-m\right)}, \end{array}$$ (2)

which follows the *F*-distribution with (m-1) and (n-m) degrees of freedom under *H*_0_ [22], where *n* = *n*_1_ + *n*_2_ +, …, + *n*_*m*_ and $\hat{\mu }=\sum_{j=1}^{m}n_{j}{\hat{\mu }}_{j}/n$. Here, ${\hat{\mu }}_{j}$ and ${\hat{\sigma }}_{j}^{2}$ are the maximum likelihood estimates (MLEs) of *μ*_*j*_ and $\sigma_{j}^{2}$, respectively, for the *j*th condition/group.

The critical region (CR) for testing *H*_0_ against *H*_1_ at the (1-*α*)100% level of significance is defined by *Pr*[*F* ≥ *F*_0_∣*H*_0_] = *α*, where *F*_0_ = *F*_*α*_(*m* − 1, *n* − *m*) is the upper 100*α*% points of the *F*-distribution with *m* − 1 and *n* − *m* degrees of freedom. *F*_0_ is also known as the cut-off point or critical value of the test. However, it is well known that the MLEs ${\hat{\theta }}_{j}=(\hat{\mu_{j}},{\hat{\sigma }}_{j}^{2})$ of $\theta_{j}=(\mu_{j},\sigma_{j}^{2})$ for *j* = 1, 2, …, *m* in Eq (2) are highly sensitive to outliers. Therefore, the identification of DE genes using classical ANOVA may produce misleading results because gene expression data are often corrupted by outliers, as previously discussed. Thus, in this paper, we consider the minimum *β*-divergence method [20, 21] to improve the robustness and efficiency of estimation in one-way ANOVA. The minimum *β*-divergence estimators ${\hat{\theta }}_{j,\beta }=({\hat{\mu }}_{j,\beta },{\hat{\sigma }}_{j,\beta }^{2})$ of the parameters $\theta_{j}=(\mu_{j},\sigma_{j}^{2})$ are computed iteratively as follows:

$$\begin{array}{c} \mu_{j,t+1}=\frac{\sum_{k=1}^{n_{j}}\phi_{\beta }\left(x_{jk}|\theta_{j,t}\right)x_{jk}}{\sum_{k=1}^{n_{j}}\phi_{\beta }\left(x_{jk}|\theta_{j,t}\right)}\; \end{array}$$ (3)

and

$$\begin{array}{c} \sigma_{j,t+1}^{2}=\frac{\sum_{k=1}^{n_{j}}\phi_{\beta }\left(x_{jk}|\theta_{j,t}\right){\left(x_{jk}-\mu_{j,t}\right)}^{2}}{{\left(\beta +1\right)}^{-1}\sum_{k=1}^{n_{j}}\phi_{\beta }\left(x_{jk}|\theta_{j,t}\right)}, \end{array}$$ (4)

where

$$\begin{array}{c} W_{\beta }\left(x_{jk}|\theta_{j}\right)=\exp\left\{-\frac{\beta }{2\sigma_{j}^{2}}{\left(x_{jk}-\mu_{j}\right)}^{2}\right\}, \end{array}$$ (5)

which we call the *β*-weight function [20, 21]. The notation ***θ***_*t*+1_ represents the update to ***θ***_*t*_ in the (*t*+1)th iteration. The robustness of these estimators is discussed in the context of influence functions in [20], and their consistency is discussed in [21]. Note that the minimum *β*-divergence estimators ${\hat{\theta }}_{j,\beta }=({\hat{\mu }}_{j,\beta },{\hat{\sigma }}_{j,\beta }^{2})$ reduce to the classical MLEs ${\hat{\theta }}_{j}=(\hat{\mu_{j}},{\hat{\sigma }}_{j}^{2})$ for *β* = 0.

Note that MLEs of a Gaussian distribution are consistent and asymptotically efficient in the absence of outliers [23]. Therefore, in this paper, an attempt is made to develop a hybrid approach in which the classical MLEs ${\hat{\theta }}_{j}$ are used in the absence of outliers and the minimum *β*-divergence estimators ${\hat{\theta }}_{j,\beta }$ (Eqs (3) and (4) are used in the presence of outliers for the estimation of ***θ***_*j*_ in one-way ANOVA. The minimum *β*-divergence method offers two approaches for unifying the robustness and efficiency of estimation in ANOVA. One method is to select the tuning parameter *β* via cross-validation (CV), as discussed in detail in a previous publication [20]. In the absence of outliers, the CV method produces *β* = 0 for the minimum *β*-divergence estimators and is thus equivalent to the classical estimators, as discussed above. In the presence of outliers, it produces *β* > 0 for the minimum *β*-divergence estimators. To develop the alternative approach, we consider the *β*-weight function (Eq (5)) with *β* = 0.2 for outlier detection. This weight function assigns smaller weights (≥ 0) to outlying/unusual observations and larger weights (≤ 1) to uncorrupted/usual observations. An outlying gene expression *x*_*jk*_ is defined based on the *β*-weight function as follows:

$$\begin{array}{ccc} W_{\beta }\left(x_{jk}|{\hat{\theta }}_{j,\beta }\right) & = & \left\{ \begin{array}{c} >\delta_{j}\text{,}\;\text{if}\;x_{jk}\;\text{is}\;\text{not}\;\text{an}\;\text{outlier}\\ \leq \delta_{j}\text{,}\;\text{if}\;x_{jk}\;\text{is}\;\text{an}\;\text{outlier,} \end{array}\right. \end{array}$$ (6)

where the threshold value *δ*_*j*_ is the *p*th quantile value of the distribution of $W_{\beta }(x_{jk}|{\hat{\theta }}_{j,\beta })$. The predicted distribution of the *β*-weight function is discussed in S1 File.

Thus, we can unify the minimum *β*-divergence estimator with MLEs for ***θ***_*j*_ in the *j*th condition as follows:

$$\begin{array}{ccc} {\hat{\theta }}_{j,\beta } & = & \left\{ \begin{array}{c} {\hat{\theta }}_{j,\beta },\;\text{if}\;\sum_{k=1}^{n_{j}}I_{\left[W_{\beta }\left(x_{jk}|{\hat{\theta }}_{j,\beta }\right)>\delta_{j}\right]}<n_{j},\\ {\hat{\theta }}_{j},\;\;\;\;\text{if}\;\sum_{k=1}^{n_{j}}I_{\left[W_{\beta }\left(x_{jk}|{\hat{\theta }}_{j,\beta }\right)>\delta_{j}\right]}=n_{j}\text{,} \end{array}\right. \end{array}$$

which leads to the formulation of the hybrid one-way ANOVA approach for the robust and efficient estimation of differential gene expression with multiple patterns with a modified F-statistic, denoted by *F*_*β*_, as given by

$$\begin{array}{c} F_{\beta }=\frac{\sum_{j=1}^{k}n_{j}{\left({\hat{\mu }}_{j,\beta }-{\hat{\mu }}_{\beta }\right)}^{2}/\left(k-1\right)}{\left[n_{1}{\hat{\sigma }}_{1,\beta }^{2}+n_{2}{\hat{\sigma }}_{2,\beta }^{2}+...+n_{m}{\hat{\sigma }}_{m,\beta }^{2}\right]/\left(n-m\right)}. \end{array}$$ (7)

To test the null hypothesis (*H*_0_) against the alternative hypothesis (*H*_1_) from the robustness perspective, we can compute *p*-values under the assumption that *F*_*β*_ approximately follows the *F*-distribution. Note that this modified F-statistic (*F*_*β*_) reduces to the classical F-statistic (Eq (2)) for *β* = 0. However, we can also compute permutation-based *p*-values to test whether *H*_0_ is true or false, as discussed in S1 File.

### 2.1 Robust Multiple Comparison Test

Multiple comparison procedures are commonly used in ANOVA after a significant omnibus test result is obtained via the *F*-test. A significant ANOVA result suggests the rejection of the global null hypothesis *H*_0_, which states that the means are identical across all groups being compared. Multiple comparison procedures are then used to determine which of the means differ significantly. In a one-way ANOVA involving *m* group means, there are *m*(*m* − 1)/2 pairwise comparisons. There are several methods for performing such a multiple comparison test. In this paper, we consider the robustification of Fisher’s *least significant difference* (LSD) test for multiple comparison tests. We reject *H*_0_ : *μ*_*i*_ = *μ*_*j*_ at the 100(1-*α*)% level of significance for all *i* ≠ *j* when $|{\hat{\mu }}_{i,\beta }-{\hat{\mu }}_{j,\beta }|>{\text{LSD}}_{\beta }$. Here, LSD_*β*_ is computed as follows:

$$\begin{array}{c} {\text{LSD}}_{\beta }=t_{\alpha /2,n-m}\sqrt{{\text{MSE}}_{\beta }}\left(\frac{1}{n_{i}}+\frac{1}{n_{j}}\right),\;\;\;\;\;\;i\neq j, \end{array}$$ (8)

where ${\text{MSE}}_{\beta }=[n_{1}{\hat{\sigma }}_{1,\beta }^{2}+n_{2}{\hat{\sigma }}_{2,\beta }^{2}+...+n_{m}{\hat{\sigma }}_{m,\beta }^{2}]/(n-m)$ and *t*_*α*/2, *n* − *m*_ is the upper (100*α*/2)% of the points of the *t*-distribution with *n* − *k* degrees of freedom. For *β* = 0, LSD_*β*_ reduces to Fisher’s (LSD) test.

## 3 Results and Discussion

We investigated the performance of the proposed method in comparison with other popular methods using both simulated and real gene expression data.

### 3.1 Performance Evaluation Based on Simulated Gene Expression Profiles

To investigate the performance of the proposed method in comparison with several popular existing methods, such as the classical parametric approach ANOVA (F-test), the nonparametric approaches SAM [10] and KW (Kruskal-Wallis test) and the empirical Bayes (EB) approaches LIMMA [13], EBarrays [5, 24], eLNN [25] and BetaEB [16], in the detection of DE (important) genes, we considered gene expression profiles simulated based on classical (ANOVA) and Bayesian (EBarrays LNN) data generation models for both small- and large-sample cases with two and multiple groups/conditions in both the absence and the presence of outlying expression, as discussed in subsections 3.1.1 and 3.1.2.

#### 3.1.1 Performance Evaluation Based on Simulated Gene Expression Profiles with *m* = 2 Conditions

To investigate the performance of the proposed method in comparison with seven popular methods (ANOVA, SAM, eLNN, LIMMA, KW, EBarrays and BetaEB), as mentioned above, for both small- and large-sample cases with *m* = 2 groups/conditions, we considered 100 datasets for both cases with sample sizes of *n*_1_ = *n*_2_ = 3,4 and 15, respectively. Each dataset for each case represented gene expression profiles for 20,000 genes, each with *n* = (*n*_1_ + *n*_2_) samples, where the expression of each gene was generated using the one-way ANOVA model described by Eq (1) for arbitrary values of (*μ*_1_, *μ*_2_) ∈ (2,5) and *σ* ^2^ = 0.05. Among the 20,000 genes represented in each dataset for each case, we generated 300 DE genes (*μ*_1_ ≠ *μ*_2_) and 19,700 EE (equally expressed) genes (*μ*_1_ = *μ*_2_). To investigate the robustness performance, we generated three types of outlying datasets from each of the original datasets by replacing at most 5% of the expression values from (*n*_1_ + *n*_2_) samples by outliers with each 5%, 10% and 75% genes, respectively. The *k*th expression in the *j*th group for a gene was corrupted by an outlier generated as follows: $x_{jk}^{*}=d+$ max(*x*_*jk*_;*k* = 1, 2, …, *n*_*j*_;*j* = 1, 2),, where *d* ∈ (5,10) is an arbitrary value.

**Fig 1:**
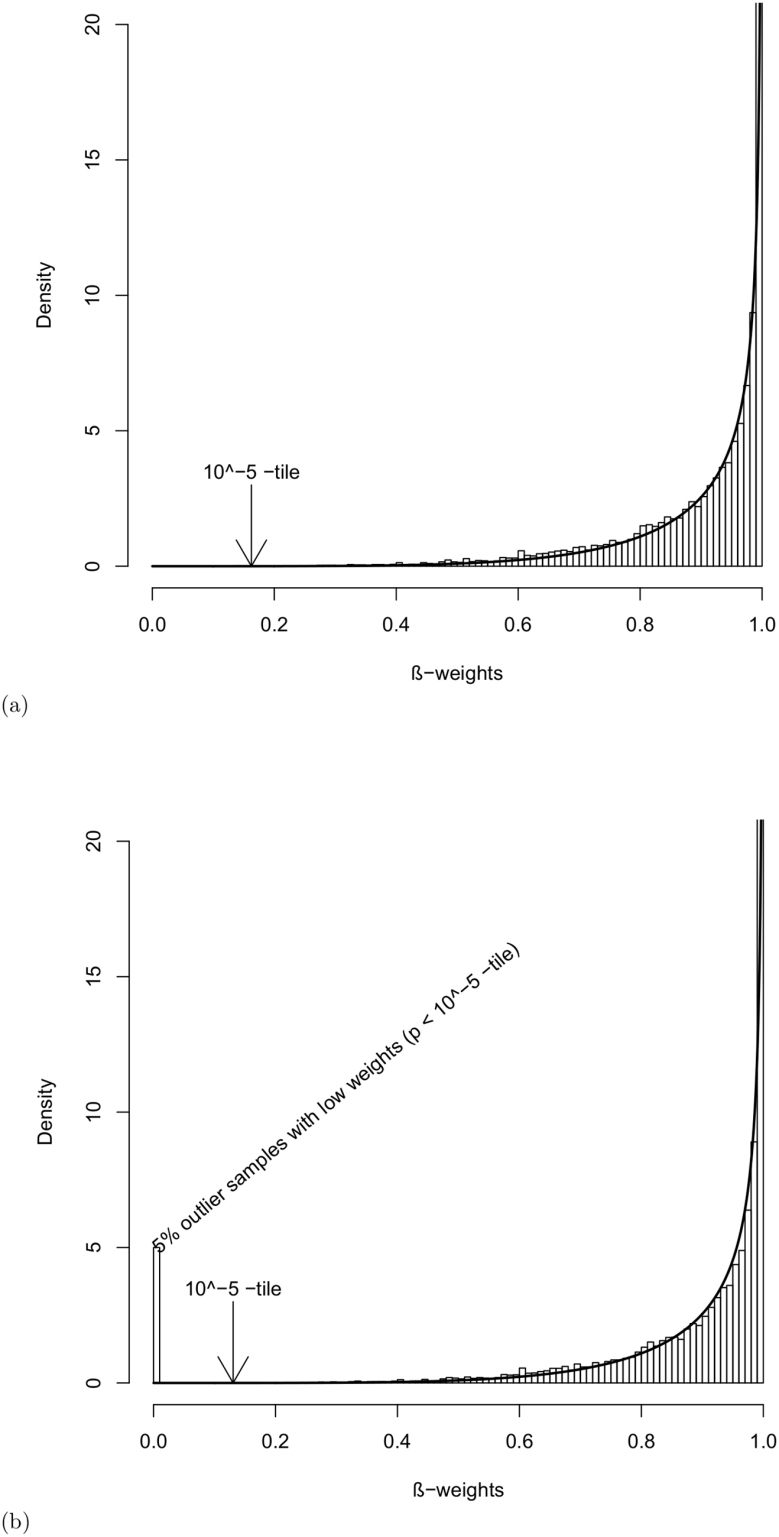
Predicted distribution of *β* weights. Predicted (solid curve) and simulated (histogram) observed distributions of the *β* weights of Eq (5): (a) without outlying gene expressions and (b) with 5% outlying gene expressions.

We first investigated the goodness of fit of the predicted distribution of the *β* weights (Eq 12 in S1 File) compared with the observed distribution of *β* weights ($W_{\beta }(\cdot |{\hat{\theta }}_{j,\beta })$). We produced both the predicted distribution (solid curve) and the observed distribution based on simulated gene expression data, assuming that $\mu_{j}={\hat{\mu }}_{j,\beta }$ and $\sigma_{j}^{2}={\hat{\sigma }}_{j,\beta }^{2}$ (Eq (1)) in both the absence and the presence of 5% or 10% outlying expressions, as shown in Fig 1a and 1b, respectively. It is clear that the predicted distributions (solid curves) in both figures reflect the corresponding observed distributions (histograms). In Fig 1b, smaller *β* weights or insignificant weights with *p*-values < 10^−5^ are evident, which correspond to outlying expressions. Note that the predicted distributions (solid curves) are identical in both figures. Therefore, we can use either of these two distributions to select the cut-off point (Eq (6)) to determine whether an expression is an outlier. Table 1 and Table A in S1 File summarize the average performance results of the eight investigated methods (ANOVA, SAM, LIMMA, eLNN, EBarrays, BetaEB, KW and Proposed) based on 100 datasets generated using a one-way ANOVA model with *m* = 2 groups/conditions and *σ* ^2^ = 0.05 for cases of both small (*n*_1_ = *n*_2_ = 3, 4) and large (*n*_1_ = *n*_2_ = 15) samples. The performance indices/measures, including the true positive rate (TPR), the false positive rate (FPR), the true negative rate (TNR), the false negative rate (FNR), the false discovery rate (FDR), the misclassification error rate (MER), the area under the ROC curve (AUC) and the partial AUC (pAUC), were calculated for each method based on their estimated top 300 DE genes, under the assumption that the other estimated genes for each method were the EE genes for each dataset (recall that each dataset contained 300 true DE genes and that the remainder were true EE genes). The values of each of these performance measures lie between 0 and 1. The method that produces the smallest values of FPR, FNR, FDR and MER and the largest values of TPR, TNR, pAUC and AUC is considered to be the best performer. The results presented in Table 1, Table A in S1 File and S1, S2, and S3 Figs clearly show the performance of all eight methods for the identification of DE genes in the absence and presence of 5% or 10% outlying expressions with 5%, 10% and 75% genes for both the small- and large-sample cases. We clearly observe that all eight methods performed similarly in the absence of outlying genes for both the small- and large-sample cases. An interesting coincidence occurs between FNR and FDR for all methods in all cases because we declared the top (1 − *p*_0_) × 100% tp be DE genes, where *p*_0_ = 0.985 is the proportion of EE genes. A similar situation was also observed in [26]. For the same reason, we also obtained almost identical results for FPR and TNR in all cases for all methods.

**Table 1:** Performance evaluation based on simulated gene expression profiles with *m* = 2 conditions/groups.

**Results for the small-sample case (n_1_ = n_2_ = 3)**																
Methods	TPR	FPR	TNR	FNR	FDR	MER	AUC	pAUC	TPR	FPR	TNR	FNR	FDR	MER	AUC	pAUC
	**Without outlying expressions**								**For 1 outlier with each of 5% genes**							
ANOVA	0.939	0.001	0.998	0.072	0.072	0.004	0.915	0.184	0.475	0.011	0.989	0.525	0.525	0.021	0.474	0.095
SAM	0.955	0.001	0.999	0.045	0.045	0.002	0.954	0.191	0.490	0.010	0.990	0.510	0.510	0.020	0.491	0.098
LIMMA	0.958	0.001	0.999	0.042	0.042	0.002	0.959	0.192	0.485	0.011	0.989	0.515	0.515	0.021	0.484	0.097
eLNN	0.932	0.001	0.999	0.068	0.068	0.003	0.931	0.186	0.372	0.013	0.987	0.627	0.627	0.025	0.371	0.074
EBarrays	0.938	0.001	0.999	0.062	0.062	0.002	0.939	0.188	0.307	0.014	0.986	0.692	0.692	0.028	0.306	0.061
BetaEB	0.938	0.001	0.999	0.062	0.062	0.002	0.937	0.188	0.940	0.001	0.999	0.060	0.060	0.002	0.941	0.188
KW	0.958	0.000	1.000	0.042	0.042	0.001	0.977	0.196	0.497	0.010	0.990	0.502	0.502	0.020	0.496	0.100
Proposed	0.939	0.001	0.998	0.072	0.072	0.004	0.915	0.184	0.936	0.001	0.998	0.064	0.064	0.004	0.914	0.182
	**For 1 outlier with each of 10% genes**								**For 1 outlier with each of 75% genes**							
ANOVA	0.318	0.014	0.986	0.682	0.682	0.027	0.318	0.063	0.087	0.019	0.981	0.912	0.912	0.036	0.086	0.018
SAM	0.323	0.014	0.986	0.677	0.677	0.027	0.324	0.064	0.087	0.019	0.981	0.912	0.912	0.036	0.086	0.018
LIMMA	0.318	0.014	0.986	0.682	0.682	0.027	0.317	0.064	0.087	0.019	0.981	0.912	0.912	0.036	0.088	0.018
eLNN	0.250	0.015	0.985	0.750	0.750	0.030	0.251	0.050	0.025	0.020	0.980	0.975	0.975	0.039	0.026	0.005
EBarrays	0.210	0.016	0.984	0.790	0.790	0.032	0.211	0.042	0.025	0.020	0.980	0.975	0.975	0.039	0.024	0.005
BetaEB	0.940	0.001	0.999	0.060	0.060	0.002	0.941	0.188	0.025	0.020	0.980	0.975	0.975	0.039	0.024	0.005
KW	0.325	0.014	0.986	0.675	0.675	0.027	0.326	0.065	0.087	0.019	0.981	0.912	0.912	0.036	0.088	0.018
Proposed	0.932	0.001	0.998	0.098	0.098	0.004	0.907	0.181	0.920	0.001	0.998	0.080	0.080	0.004	0.887	0.177
**Results for the large-sample case (**n_1_ = n_2_ = 15**)**																
Methods	TPR	FPR	TNR	FNR	FDR	MER	AUC	pAUC	TPR	FPR	TNR	FNR	FDR	MER	AUC	pAUC
**Without outlying expressions**								**For 1 or 2 outliers with each of 5% genes**							
ANOVA	0.971	0.002	0.998	0.026	0.026	0.001	0.972	0.195	0.560	0.009	0.991	0.440	0.440	0.018	0.561	0.112
SAM	0.978	0.000	1.000	0.022	0.022	0.001	0.979	0.196	0.613	0.008	0.992	0.388	0.388	0.015	0.612	0.122
LIMMA	0.978	0.000	1.000	0.022	0.022	0.001	0.977	0.196	0.562	0.009	0.991	0.438	0.438	0.018	0.563	0.112
eLNN	0.975	0.001	0.999	0.025	0.025	0.001	0.974	0.195	0.850	0.003	0.997	0.150	0.150	0.006	0.849	0.170
EBarrays	0.973	0.001	0.999	0.028	0.028	0.001	0.974	0.195	0.562	0.009	0.991	0.438	0.438	0.018	0.561	0.112
BetaEB	0.973	0.001	0.999	0.028	0.028	0.001	0.975	0.195	0.973	0.001	0.999	0.028	0.028	0.001	0.972	0.195
KW	0.973	0.001	0.999	0.028	0.028	0.001	0.973	0.194	0.911	0.002	0.998	0.089	0.089	0.008	0.801	0.160
Proposed	0.971	0.002	0.998	0.026	0.026	0.001	0.973	0.195	0.975	0.001	0.999	0.025	0.025	0.001	0.976	0.195
**For 1 or 2 outliers with each of 10% genes**								**For 1 or 2 outliers with each of 75% genes**							
ANOVA	0.420	0.012	0.988	0.580	0.580	0.023	0.420	0.084	0.312	0.014	0.986	0.688	0.688	0.028	0.311	0.062
SAM	0.497	0.010	0.990	0.502	0.502	0.020	0.497	0.099	0.347	0.013	0.987	0.652	0.652	0.026	0.347	0.069
LIMMA	0.425	0.012	0.988	0.575	0.575	0.023	0.425	0.085	0.347	0.013	0.987	0.652	0.652	0.026	0.348	0.069
eLNN	0.885	0.002	0.998	0.115	0.115	0.005	0.885	0.177	0.855	0.003	0.997	0.145	0.145	0.006	0.856	0.171
EBarrays	0.427	0.012	0.988	0.573	0.573	0.023	0.427	0.085	0.228	0.016	0.984	0.772	0.772	0.031	0.227	0.045
BetaEB	0.973	0.001	0.999	0.028	0.028	0.001	0.973	0.195	0.225	0.016	0.984	0.775	0.775	0.031	0.224	0.045
KW	0.851	0.004	0.996	0.149	0.149	0.008	0.807	0.161	0.808	0.009	0.991	0.192	0.192	0.002	0.936	0.187
Proposed	0.973	0.001	0.999	0.028	0.028	0.001	0.973	0.195	0.978	0.000	1.000	0.022	0.022	0.001	0.979	0.196

Average performance results of eight methods (ANOVA, SAM, LIMMA, eLNN, EBarrays, BetaEB, KW and Proposed) based on 100 datasets generated using a one-way ANOVA model with *m* = 2 groups/conditions and *σ* ^2^ = 0.05 for both sample sizes n1 = n2 = 3 and n1 = n2 = 15. Each dataset for each case contained 300 true DE genes, and the remainder were 19700 true EE genes. The performance indices/measures TPR, FPR, TNR, FNR, FDR, MER and AUC were calculated for each method based on the top 300 estimated DE genes, under the assumption that the other estimated genes in each dataset for each case were EE genes for each method. The performance measure ‘pAUC’ was calculated at FPR = 0.2 for each method and for each dataset.

However, in the presence of 1 or 2 outlying expressions with 5%, 10% and 75% genes for the small- or large-sample or both sample cases, four methods (eLNN, KW, BetaEB and Proposed) exhibited better performance on average than the other four methods (ANOVA, SAM, EBarrays and LIMMA) because the former four methods produce larger values of TPR, AUC and pAUC and smaller values of FNR, FDR and MER compared to the other four methods. Two methods (KW and eLNN) showed slightly good performance for the large-sample case, but they were sensitive to outliers for the small-sample case. The empirical Bayes approach (BetaEB) exhibited good performance for both the small- and large-sample cases in the presence of outlying expressions with 5% and 10% genes, but it exhibited weak performance in the presence of outlying expressions in 75% genes. Thus, the robust BetaEB method and the proposed method exhibited better performance than the other six methods in presence of at most 50% outlying genes (although we provided results with 5%, 10% and 75% outlying genes only) with 5% outliers for each outlying gene. The BetaEB approach appears to be slightly better than the proposed method for the small-sample case, whereas the proposed method exhibited considerably better performance than the BetaEB method for both the small- and large-sample cases in the presence of more than 50% outlying genes. We also observed similar results when we generated gene expression profiles based on a Bayesian (EBarrays LNN) data generating model considering all aspects of the ANOVA model (Table B in S1 File).

#### 3.1.2 Performance Evaluation Based on Simulated Gene Expression Profiles with *m* ≥ 3 Conditions with Multiple Patterns

To investigate the performance of the proposed method in comparison with the other methods for *m* = 4 conditions with multiple patterns for both small- and large-sample cases, we tested these methods on 100 datasets for sample sizes of *n*_1_ = *n*_2_ = *n*_3_ = *n*_4_ = 3 and 15, respectively. Each dataset for each case represented gene expression profiles for 20,000 genes, each with *n* = (*n*_1_ + *n*_2_ + *n*_3_ + *n*_4_) samples, where the expression of each gene was generated using the one-way ANOVA model described by Eq (1) for arbitrary values of (*μ*_1_, *μ*_2_, *μ*_3_, *μ*_4_) ∈ (3,5) and *σ* ^2^ = 0.05. We generated 19,700 EE genes (*μ*_1_ = *μ*_2_ = *μ*_3_ = *μ*_4_) and 300 DE genes with patterns such as (i) *μ*_1_ = *μ*_2_ ≠ *μ*_3_ = *μ*_4_, (ii) *μ*_1_ = *μ*_2_ ≠ *μ*_3_ ≠ *μ*_4_, and (iii) *μ*_1_ ≠ *μ*_2_ ≠ *μ*_3_ ≠ *μ*_4_ for each dataset for each case. To investigate the robustness performance for m = 4 conditions with multiple patterns, we generated two types of outlying datasets from each of the original datasets by replacing at most 5% of the expression values from (*n*_1_ + *n*_2_ + *n*_3_ + *n*_4_) samples by outliers with each 5% and 10% genes, respectively. An expression of (*x*_*jk*_;*k* = 1, 2, …, *n*_*j*_;*j* = 1,2,3,4) for a gene was corrupted by an outlier generated as follows: $x_{jk}^{*}=d+$ max(*x*_*jk*_;*k* = 1, 2, …, *n*_*j*_;*j* = 1, 2, 3, 4), where *d* ∈ (5,10) is an arbitrary value.

In this study, we investigated the performance of the proposed method in comparison with only four popular methods (ANOVA, SAM, LIMMA and KW) because the robust BetaEB approach is not applicable for multiple patterns of expression because of its computational complexity. Table 2 shows that all the methods, except KW, produce almost identical values of FDR, AUC and pAUC in the absence of outlying genes for the small-sample case. KW was found to have considerably lower values of AUC and pAUC and a larger value of FDR for multiple groups/conditions (m = 4) in the small-sample case. Additionally, it exhibits greater sensitivity than the other methods (ANOVA, SAM, LIMMA and proposed) in the presence of outlying genes. However, all of the methods, including KW, perform similarly in the absence of outlying genes for the large-sample case. In this case, the performance of KW is better than that of the other three methods (ANOVA, SAM and LIMMA) in the presence of outlying genes, but it is slightly weaker than that of the proposed method. Thus, in all cases, the proposed method exhibited performance similar to that of the other approaches in the absence of outliers and better performance in the presence of outliers with lower FDR values and higher AUC and pAUC values. We also investigated the performance of the proposed method in comparison with the same four methods (ANOVA, SAM, LIMMA and KW) for *m* = 4 conditions with FDR and with the family-wise error rate (FWER) fixed to 1% using adjusted *p*-values based on the Benjamini-Hochberg (BH) and Bonferroni corrections, respectively. Tables 3 and 4 present the average TPR using *p*-values and adjusted *p*-values at the 1% level of significance for both the small- and large-sample cases, respectively. The results shown in brackets () represent the average FPR. Table 3 also shows that three methods (ANOVA, LIMMA and proposed) are strong enough to achieve higher TPR (≥ 80%) than SAM and KW using both raw *p*-values and adjusted *p*-values in the absence of outlying genes. The SAM obtains a lower TPR than the other three methods (ANOVA, LIMMA and proposed) when controlling FWER by Bonferroni corrections. The KW also shows that there are no DE genes in the absence of outlying genes for the small-sample case (Table 3). As an example, from Table 3, we observe that ANOVA, SAM, LIMMA, KW and the proposed methods obtained TPRs of 0.944 (0.012), 0.947 (0.003), 0.955 (0.009), 0.000 (0.000) and 0.944 (0.012) based on the true set of 300 DE genes in the absence of outlying genes, FPR shown in parenthesis, on average, respectively.

**Table 2:** Performance evaluation based on simulated gene expression profiles with *m* ≥ 3 conditions with multiple patterns.

Methods					
PM/PI	ANOVA	SAM	LIMMA	KW	Proposed
	**Average performance in the small-sample case (**n_1_ = n_2_ = n_3_ = n_4_ = 3**)**				
	**Without outlying expressions**				
FDR	0.0967	0.0915	0.0833	0.8600	0.0967
AUC	0.9012	0.9089	0.9105	0.1394	0.9012
pAUC	0.1816	0.1825	0.1854	0.0274	0.1816
	**For a single outlying expression with each of 5% genes**				
FDR	0.5467	0.4400	0.5400	0.9033	0.1067
AUC	0.4533	0.5600	0.4599	0.0962	0.8933
pAUC	0.0906	0.1120	0.0919	0.0188	0.1786
	**For a single outlying expression with each of 10% genes**				
FDR	0.6967	0.7633	0.6933	0.9200	0.1100
AUC	0.3033	0.2362	0.3066	0.0796	0.8900
pAUC	0.0606	0.0469	0.0613	0.0156	0.1780
	**Average performance in the large-sample case (**n_1_ = n_2_ = n_3_ = n_4_ = 15**)**				
	**Without outlying expressions**				
FDR	0.0200	0.0200	0.0200	0.0200	0.0233
AUC	0.9800	0.9800	0.9800	0.9800	0.9767
pAUC	0.1960	0.1960	0.1960	0.1960	0.1953
	**For 1-2 outlying expression in each 5% genes**				
FDR	0.3833	0.3367	0.3800	0.0567	0.0233
AUC	0.6165	0.6633	0.6198	0.9433	0.9767
pAUC	0.1231	0.1326	0.1238	0.1886	0.1953
	**For 1-2 outlying expression in each 10% genes**				
FDR	0.4933	0.4633	0.4267	0.0833	0.0233
AUC	0.5063	0.5366	0.5727	0.9166	0.9767
pAUC	0.1010	0.1073	0.1140	0.1833	0.1953

Average performance results for five methods (ANOVA, SAM, LIMMA, KW and Proposed) based on 100 datasets generated using a one-way ANOVA model for both small- and large-sample cases with *m* = 4 groups/conditions and *σ* ^2^ = 0.05. The performance measure/index (PM/PI) FDR was calculated for each method based on the top 300 estimated DE genes, under the assumption that the other estimated genes were EE genes for each dataset (recall that each dataset contained 300 true DE genes and the remainder were EE genes). The performance measure ‘pAUC’ was calculated at FPR = 0.2 for each method and for each dataset.

**Table 3:** Performance evaluation based on simulated gene expression profiles with *m* ≥ 3 conditions with multiple patterns based on raw *p*-values and adjusted *p*-values (controlling FDR and FWER) at 1% for a small sample size.

METHODS	Without outlying expressions	With 5% outlying expressions in 5% genes	With 5% outlying expressions in 10% genes
**Using raw *p*-values**			
*TPR.ANOVA*	0.944	0.467	0.314
	(0.012)	(0.012)	(0.011)
*TPR.SAM*	0.947	0.553	0.220
	(0.003)	(0.001)	(0.006)
*TPR.LIMMA*	0.955	0.471	0.315
	(0.009)	(0.012)	(0.012)
*TPR.KW*	0.000	0.000	0.000
	(0.000)	(0.000)	(0.000)
*TPR.Proposed*	0.944	0.941	0.939
	(0.012)	(0.012)	(0.013)
**Using adjusted *p*-values with Benjamini-Hochberg (BH) procedure (controlling FDR at 1%)**			
*TPR.ANOVA.BH*	0.893	0.431	0.284
	(0.000)	(0.000)	(0.000)
*TPR.SAM.BH*	0.440	0.390	0.000
	(0.000)	(0.000)	(0.000)
*TPR.LIMMA.BH*	0.905	0.438	0.291
	(0.000)	(0.000)	(0.000)
*TPR.KW.BH*	0.000	0.000	0.000
	(0.000)	(0.000)	(0.000)
*TPR.Proposed.BH*	0.893	0.873	0.875
	(0.000)	(0.000)	(0.000)
**Using adjusted *p*-values with Bonferroni correction procedure (controlling FWER at 1%)**			
*TPR.ANOVA.Bonf*	0.891	0.383	0.256
	(0.000)	(0.000)	(0.000)
*TPR.SAM.Bonf*	0.623	0.328	0.222
	(0.000)	(0.000)	(0.000)
*TPR.LIMMA.Bonf*	0.901	0.399	0.264
	(0.000)	(0.000)	(0.000)
*TPR.KW.Bonf*	0.000	0.000	0.000
	(0.000)	(0.000)	(0.000)
*TPR.Proposed.Bonf*	0.891	0.890	0.888
	(0.000)	(0.000)	(0.000)

Average performance results for five methods (ANOVA, SAM, LIMMA, KW and Proposed) based on 100 datasets generated using a one-way ANOVA model for the small-sample (*n*_1_ = *n*2 = *n*_3_ = *n*_4_ = 3) case with *m* = 4 groups/conditions and *σ* ^2^ = 0.05, where each dataset contained 300 true genes and the remainder were 19700 true EE genes. The values represent the average TPRs based on raw *p*-values and adjusted *p*-values for the ANOVA, SAM, LIMMA, KW and Proposed methods in both the absence and the presence of outlying expressions. The value within the bracket () indicates the average FPRs. The adjusted *p*-values were calculated using Benjamini-Hochberg (BH) and Bonferroni correction methods.

**Table 4:** Performance evaluation based on simulated gene expression profiles with *m* ≥ 3 conditions with multiple patterns based on raw *p*-values and adjusted *p*-values (controlling FDR and FWER) at 1% for a large sample size.

METHODS	Without outlying expressions	With 5% outlying expressions in 5% genes	With 5% outlying expressions in 10% genes
**Using raw *p*-values**			
*TPR.ANOVA*	0.981	0.631	0.503
	(0.010)	(0.009)	(0.009)
*TPR.SAM*	0.980	0.841	0.719
	(0.004)	(0.032)	(0.056)
*TPR.LIMMA*	0.983	0.635	0.508
	(0.010)	(0.009)	(0.009)
*TPR.KW*	0.982	0.975	0.975
	(0.008)	(0.008)	(0.008)
*TPR.Proposed*	0.983	0.981	0.981
	(0.037)	(0.036)	(0.036)
**Using adjusted *p*-values with Benjamini-Hochberg (BH) procedure (controlling FDR at 1%)**			
*TPR.ANOVA.BH*	0.975	0.522	0.381
	(0.000)	(0.000)	(0.000)
*TPR.SAM.BH*	0.957	0.602	0.450
	(0.000)	(0.000)	(0.000)
*TPR.LIMMA.BH*	0.977	0.525	0.386
	(0.000)	(0.000)	(0.000)
*TPR.KW.BH*	0.972	0.872	0.855
	(0.000)	(0.000)	(0.000)
*TPR.Proposed.BH*	0.977	0.968	0.975
	(0.002)	(0.002)	(0.002)
**Using adjusted *p*-values with Bonferroni correction procedure (controlling FWER at 1%)**			
*TPR.ANOVA.Bonf*	0.965	0.485	0.345
	(0.000)	(0.000)	(0.000)
*TPR.SAM.Bonf*	0.937	0.477	0.333
	(0.000)	(0.000)	(0.000)
*TPR.LIMMA.Bonf*	0.970	0.485	0.345
	(0.000)	(0.000)	(0.000)
*TPR.KW.Bonf*	0.941	0.581	0.469
	(0.000)	(0.000)	(0.000)
*TPR.Proposed.Bonf*	0.969	0.955	0.968
	(0.000)	(0.000)	(0.000)

Average performance results for five methods (ANOVA, SAM, LIMMA, KW and Proposed) based on 100 datasets generated using a one-way ANOVA model for the large-sample (*n*_1_ = *n*2 = *n*_3_ = *n*_4_ = 15) case with *m* = 4 groups/conditions and *σ* ^2^ = 0.05, where each dataset contained 300 true genes and the remainder were 19700 true EE genes. The values represent the average TPRs based on raw *p*-values and adjusted *p*-values for the ANOVA, SAM, LIMMA, KW and Proposed methods in both the absence and the presence of outlying expressions. The value within the bracket () indicates the average FPRs. The adjusted *p*-values were calculated using Benjamini-Hochberg (BH) and Bonferroni correction methods.

However, in the presence of outlying genes, Tables 3 and 4 both show that most of the methods, except the proposed method, are unable to achieve high TPRs for both sample cases. Interestingly, we observed that all methods, including SAM and KW, perform well for the large-sample case in the absence of outlying genes, whereas we observed a loss of efficiency of SAM and KW for the small-sample case. The nonparametric approach KW is more efficient than the other three methods (ANOVA, SAM and LIMMA) in the presence of outliers in different proportions of genes for the large-sample case (Table 4). Therefore, we observed that the proposed method performed similarly in the absence of outliers, but it exhibited the best performance in the presence of outliers compared to all other methods in all cases of both sample sizes (Tables 3 and 4).

The results presented in Tables 2, 3 and 4 were calculated under the global null hypothesis (*H*_0_) of no differential gene expressions in the *m* = 4 conditions. However, after rejection of the global *H*_0_, a multiple comparison test was required to identify the pattern of differential gene expression. Tables 5 and 6 present the adjusted *p*-values of the multiple comparison tests for ANOVA, LIMMA, KW and the proposed method based on the datasets corresponding to the true patterns *μ*_1_ = *μ*_2_ ≠ *μ*_3_ = *μ*_4_ and *μ*_1_ = *μ*_2_ ≠ *μ*_3_ ≠ *μ*_4_, respectively. Both tables indicate that all four methods (ANOVA, LIMMA, KW and Proposed) were successful in identifying the correct patterns of differential gene expression in the absence of outlying expressions, whereas in the presence of a few outliers (5%), the proposed method exhibited superior performance in identifying the correct patterns. For example, in the absence of outlying genes, all four methods (ANOVA, LIMMA, KW and Proposed) provided larger *p*-values, such as 0.9379, 1.000, 0.8808 and 0.5537, respectively (Table 5), because we considered no difference between group 1 and group 2 in the pattern of a gene. Among them, the LIMMA performed better (*p*-value = 1) than the other approaches in obtaining the true difference between group 1 and group 2 of the pattern. In the case of group 1 and group 3, all four methods detected the true difference between group 1 and group 3 of the pattern, but among them, the ANOVA, LIMMA and proposed methods performed better than the KW method in obtaining the true pattern. However, two (ANOVA and LIMMA) of the four methods failed to detect the true patterns in the presence of 5% outliers in the previous expression, as shown in Tables 5 and 6, respectively. The nonparametric approach KW performed more poorly than these two methods in detecting the true patterns with larger p-values, even in the absence of outlying genes for the small-sample case, but better for the large-sample case with multiple groups in both the absence and presence of outlying genes, whereas the proposed method remained similar, as previously mentioned, to the case where outliers were absent in both sample sizes.

**Table 5:** Performance evaluation in multiple comparison tests using four methods (ANOVA, LIMMA, KW and Proposed) for a small sample size.

	*μ*_1_	*μ*_2_	*μ*_3_
Without outlying expressions			
*μ*_2_	(0.9379)	-	-
	{1.0000}	-	-
	[0.8808]	-	-
	⟨0.5537⟩	-	-
*μ*_3_	(0.0003)	(0.0004)	-
	{0.0000}	{0.0000}	-
	[0.0364]	[0.0419]	-
	⟨0.0001⟩	⟨0.0001⟩	-
*μ*_4_	(0.0003)	(0.0006)	(0.9110)
	{0.0000}	{0.0001}	{1.0000}
	[0.0396]	[0.0450]	[0.8796]
	⟨0.0001⟩	⟨0.0002⟩	⟨0.5166⟩
With 5% outlying expressions			
*μ*_2_	(0.9343)	-	-
	{1.0000}	-	-
	[0.7964]	-	-
	⟨0.5498⟩	-	-
*μ*_3_	(0.3865)	(0.4527)	-
	{0.6605}	{0.6610}	-
	[0.4104]	[0.4738]	-
	⟨0.0002⟩	⟨0.0002⟩	-
*μ*_4_	(0.3759)	(0.4393)	(0.9026)
	{0.6057}	{0.6313}	{1.0000}
	[0.3717]	[0.4564]	[0.8553]
	⟨0.0002⟩	⟨0.0002⟩	⟨0.5163⟩

Average *p*-values for multiple comparison tests using four methods (ANOVA, LIMMA, KW and Proposed) based on 100 sets of expressions for a DE gene with the pattern *μ*_1_ = *μ*_2_ ≠ *μ*_3_ = *μ*_4_ for small samples of size (n1 = n2 = n3 = n4 = 3) with *m* = 4 conditions and *σ* ^2^ = 0.05. The expression profiles for the gene were generated using a one-way ANOVA model. The *p*-value was calculated for each method and each dataset. A larger *p*-value indicates equality between the mean expressions of two groups. The bracket types (), {}, [] and ⟨⟩ indicate the average gene *p*-values obtained using ANOVA, LIMMA, KW and the proposed methods, respectively.

**Table 6:** Performance evaluation in multiple comparison tests using four methods (ANOVA, LIMMA, KW and Proposed) for a large sample size.

	*μ*_1_	*μ*_2_	*μ*_3_
Without outlying expressions			
*μ*_2_	(0.9944)	-	-
	{1.0000}	-	-
	[0.9118]	-	-
	⟨0.5709⟩	-	-
*μ*_3_	(0.0000)	(0.0000)	-
	{0.0000}	{0.0000}	-
	[0.0000]	[0.0000]	-
	⟨0.0000⟩	⟨0.0000⟩	-
*μ*_4_	(0.0000)	(0.0000)	(0.0000)
	{0.0000}	{0.0000}	{0.0000}
	[0.0000]	[0.0000]	[0.0000]
	⟨0.0000⟩	⟨0.0000⟩	⟨0.0000⟩
With 5% outlying expressions			
*μ*_2_	(0.9213)	-	-
	{1.0000}	-	-
	[0.8504]	-	-
	⟨0.5007⟩	-	-
*μ*_3_	(0.4452)	(0.4481)	-
	{0.7440}	{0.7639}	-
	[0.0003]	[0.0001]	-
	⟨0.0000⟩	⟨0.0000⟩	-
*μ*_4_	(0.2645)	(0.2219)	(0.6475)
	{0.4775}	{0.4008}	{0.9199}
	[0.0000]	[0.0000]	[0.0026]
	⟨0.0000⟩	⟨0.0000⟩	⟨0.0000⟩

Average *p*-values for multiple comparison tests using four methods (ANOVA, LIMMA, KW and Proposed) based on 100 sets of expressions for a DE gene with the pattern *μ*_1_ = *μ*_2_ ≠ *μ*_3_ ≠ *μ*_4_ for large samples of size (n1 = n2 = n3 = n4 = 15) with *m* = 4 conditions and *σ* ^2^ = 0.05. The expression profiles for the gene were generated using a one-way ANOVA model. The *p*-value was calculated for each method and each dataset. A larger *p*-value indicates equality between the mean expressions of two groups. The bracket types (), {}, [] and ⟨⟩ indicate the average gene *p*-values obtained using ANOVA, LIMMA, KW and the proposed methods, respectively.

To investigate the pattern-detection performance of the proposed method in comparison to the others, we generated 300 DE genes among the 20,000 genes in the dataset for m = 4 conditions with different patterns for the sample size (n1 = n2 = n3 = n4 = 6) with a 2-fold change in expression between the groups. Then, we first applied four methods (ANOVA, LIMMA, KW and Proposed) to test the null hypothesis of no differential expression among four groups for each gene. If the test was rejected, then we applied the multiple comparison test for pattern detection of gene expression. Table 7 presents the multiple comparison results, where the values presented in the form {x, x, x, x} indicate the numbers of downregulated (DR) or upregulated (UR) differentially expressed (DE) genes estimated using ANOVA, LIMMA, KW and the proposed methods, respectively. This table also describes the true patterns of the DE genes in the various groups. We observe that all four methods exhibited nearly identical performance in the absence of outlying genes; however, in the presence of a single outlying expression in each of 10% genes, the proposed method exhibited far superior performance compared to the other methods.

**Table 7:** Performance evaluation in pairwise comparison tests using four methods (ANOVA, LIMMA, KW and Proposed) for the small-sample case.

**Adjusted *p*-value < 0.05**						
Pair of groups	Predicted DR ${\left(\text{log}\frac{{\hat{\mu }}_{i}}{{\hat{\mu }}_{j}}<-1\right)}^{a}$	True DR	Correctly predicted DR	Predicted UR ${\left(\text{log}\frac{{\hat{\mu }}_{i}}{{\hat{\mu }}_{j}}>+1\right)}^{a}$	True UR	Correctly predicted UR
	**Without outlying expressions**					
G1 vs. G2	{49, 51, 50, **49**}	50	{49, 50, 50, **49**}	{101, 100, 98, **101**}	100	{100, 99, 98, **100**}
G1 vs. G3	{130, 129, 130, **130**}	130	{130, 129, 130, **130**}	{99, 102, 101, **99**}	100	{99, 100, 100, **99**}
G1 vs. G4	{50, 49, 49, **50**}	50	{50, 49, 48, **50**}	{101, 100, 99, **101**}	100	{100, 99, 99, **100**}
G2 vs. G3	{100, 101, 102, **100**}	100	{99, 100, 100, **99**}	{20, 19, 20, **20**}	20	{20, 19, 20, **20**}
G2 vs. G4	{51, 49, 47, **51**}	50	{50, 49, 47, **50**}	{49, 50, 49, **49**}	50	{49, 50, 49, **49**}
G3 vs. G4	{50, 50, 51, **50**}	50	{49, 48, 50, **49**}	{132, 128, 129, **132**}	130	{130, 128, 129, **130**}
	**With a single outlying expression in 10% genes**					
G1 vs. G2	{37, 31, 41, **49**}	50	{37, 31, 41, **49**}	{83, 74, 84, **100**}	100	{83, 74, 84, **100**}
G1 vs. G3	{107, 90, 112, **130**}	130	{107, 90, 112, **130**}	{83, 76, 84, **99**}	100	{83, 76, 84, **99**}
G1 vs. G4	{43, 38, 43, **50**}	50	{43, 38, 43, **50**}	{83, 69, 83, **101**}	100	{83, 69, 83, **100**}
G2 vs. G3	{84, 69, 87, **100**}	100	{84, 69, 87, **99**}	{15, 12, 16, **20**}	20	{15, 12, 16, **20**}
G2 vs. G4	{43, 36, 43, **51**}	50	{43, 36, 43, **50**}	{37, 27, 43, **49**}	50	{37, 27, 43, **49**}
G3 vs. G4	{43, 37, 43, **50**}	50	{43, 37, 43, **49**}	{107, 87, 117, **131**}	130	{107, 87, 117, **130**}

We generated 300 DE genes out of 20,000 total genes for *m* = 4 conditions with different patterns for a small-sample case (n1 = n2 = n3 = n4 = 6) and *σ* ^2^ = 0.05, with a 2-fold change in expression between the groups, to investigate the pattern-detection performance of the proposed method in comparison with the others. The values reported in the form {x, x, x, x} in this table represent the numbers of downregulated (DR) or upregulated (UR) differentially expressed (DE) genes estimated by the ANOVA, LIMMA, KW and proposed (Bold) methods, respectively. ^*a*^Note that $\text{log}\frac{{\hat{\mu }}_{i}}{{\hat{\mu }}_{j}}<-1$ indicates significant 2-fold downregulation and $\text{log}\frac{{\hat{\mu }}_{i}}{{\hat{\mu }}_{j}}>+1$ indicates significant 2-fold upregulation.

### 3.2 Performance Evaluation Based on Real Gene Expression Profiles

We considered three publicly available microarray gene expression datasets in the analyses presented in subsections 3.2.1, 3.2.2 and 3.2.3 to evaluate the performance of the proposed method in comparison with several popular methods, as discussed above. These three datasets are (i) the platinum spike gene expression dataset [27], (ii) the colon cancer gene expression dataset [28] and (iii) the pancreatic cancer gene expression dataset [29]. All three datasets were generated using Affymetrix technology.

#### 3.2.1 Analysis of the Platinum Spike Gene Expression Dataset with *m* = 2 Conditions

This dataset was previously analyzed in [4, 27]. It consists of 18 spike-in samples (9 controls versus 9 test cases). We downloaded this dataset from the GEO website under the accession number GSE21344. We also downloaded the designated fold change (FC) dataset associated with the probes from www.biomedcentral.com/content/supplementary/1471-2105-11-285-s5.txt. After pre-processing (using RMA) and filtering the dataset, we obtained gene expressions with 18707 probes, among which 1944 probes are known as the designated DE genes under spiked-in fold changes of 0.25, 0.28, 0.40, 0.66, 0.83, 1.5, 1.7, 2, 3 and 3.5. In our analysis, we consider these designated DE genes as designated ‘DE gene-set’ and the rest of the genes as designated ‘EE gene-set’ for performance evaluation of the proposed method in a comparison with the other seven methods (ANOVA, SAM, eLNN, LIMMA, KW, EBarrays, BetaEB). We applied all eight methods to the dataset to identify the DE genes. We considered the estimated top 1944 genes for each method and crossed with the designated ‘DE gene-set’ to calculate the same summary statistics (TPR, TNR, FPR, FNR, FDR, MER, AUC and pAUC) for performance evaluation as used for the simulation studies. Table 8 shows that all the methods produce similar results with the original spike dataset. Although the performance of all methods appears to be similar for this dataset, the *β*-weight function of the proposed method detected 9% (= 1684) genes as outlying genes, where 1634 outlying genes belonged to the designated EE gene-set and the remaining 50 genes belonged to the designated DE genes. Outlier genes are indicated with a red color in S4(a)–S4(c) Fig. Both EE and DE outlying genes are distributed with respect to the interval of estimated *p*-values or (1-posterior probabilities), as shown in S4(d)–S4(e) Fig for each method. We observed that the proposed method detected the largest number of designated EE (233) and DE (40) outlying genes with *p*-values of less than 0.05. To investigate the reason for why the proposed method detected the largest number of designated EE outlying genes with *p*-values of less than 0.05 compared to the other approaches, we present the M-A plot based on group medians in S4F Fig. In the M-A plot, the marker (⋅) with a gray color represents all genes, the marker (°) with a green color represents designated DE genes, the marker (*) with a blue color represents designated EE outlying genes, and the marker (*) with a red color represents designated DE outlying genes. We clearly observed that some designated DE genes belong to the zero line (EE line); however, no designated EE outlying genes belong to the zero line (EE line). Therefore, the outlying 233 designated EE genes detected by the proposed method as DE genes with *p*-values of less than 0.05 should belong to the designated DE gene-set. Thus, the robust methods (KW, BetaEB and Proposed) did not perform better than the classical methods (ANOVA, SAM, LIMMA, eLNN and EBarrays), even for the dataset containing outlying genes. To show the outlier effects on the methods, we corrupted low label expressions of 200 designated DE genes by a single outlier larger than the maximum value of expressions. Then, we applied all methods again and evaluated the performance, as shown in Table 8. We found that the proposed and BetaEB methods remained almost unchanged for all the summary statistics, as previously calculated in the case of the original expression, whereas all of the other methods decreased TPR, AUC, and pAUC and increased FNR, FDR and MER significantly. Thus, the application of robust methods would be better than classical methods for real gene expression data analysis.

**Table 8:** Performance evaluation based on Spike gene expression profiles with 2 conditions for the sample case (n_1_ = n_2_ = 9).

**Original expression**								
Methods	TPR	FPR	TNR	FNR	FDR	MER	AUC	pAUC
ANOVA	0.8189	0.0210	0.9790	0.1811	0.1811	0.0376	0.8165	0.1613
SAM	0.8338	0.0193	0.9807	0.1662	0.1662	0.0345	0.8319	0.1648
LIMMA	0.8297	0.0197	0.9803	0.1703	0.1703	0.0354	0.8283	0.1645
eLNN	0.8071	0.0224	0.9776	0.1929	0.1929	0.0401	0.8057	0.1600
EBarrays	0.8292	0.0198	0.9802	0.1708	0.1708	0.0355	0.8275	0.1641
BetaEB	0.8066	0.0201	0.9799	0.1934	0.1934	0.0360	0.8247	0.1634
KW	0.8097	0.0221	0.9779	0.1903	0.1903	0.0396	0.8053	0.1576
Proposed	0.8098	0.0195	0.9805	0.1902	0.1902	0.0348	0.8225	0.1647
**Including single outlier in each of 200 genes from designated set of 1944 genes**								
ANOVA	0.7618	0.0276	0.9724	0.2382	0.2382	0.0495	0.7592	0.1498
SAM	0.7731	0.0263	0.9737	0.2269	0.2269	0.0471	0.7704	0.1519
LIMMA	0.7644	0.0273	0.9727	0.2356	0.2356	0.0490	0.7623	0.1508
eLNN	0.7757	0.0260	0.9740	0.2243	0.2243	0.0466	0.7673	0.1467
EBarrays	0.7567	0.0282	0.9718	0.2433	0.2433	0.0506	0.7554	0.1500
BetaEB	0.8061	0.0202	0.9798	0.1939	0.1939	0.0361	0.8242	0.1633
KW	0.7536	0.0286	0.9714	0.2464	0.2464	0.0512	0.7490	0.1461
Proposed	0.8090	0.0199	0.9801	0.1910	0.1910	0.0363	0.8215	0.1636

We considered the estimated top 1944 genes for each method and then crossed with the designated ‘DE gene-set’ to calculate the summary statistics (TPR, TNR, FPR, FNR, FDR, MER, AUC and pAUC) for performance evaluation in the Spike gene expression profiles.

#### 3.2.2 Analysis of the Colon Cancer Gene Expression Dataset with *m* = 2 Conditions

**Fig 2:**
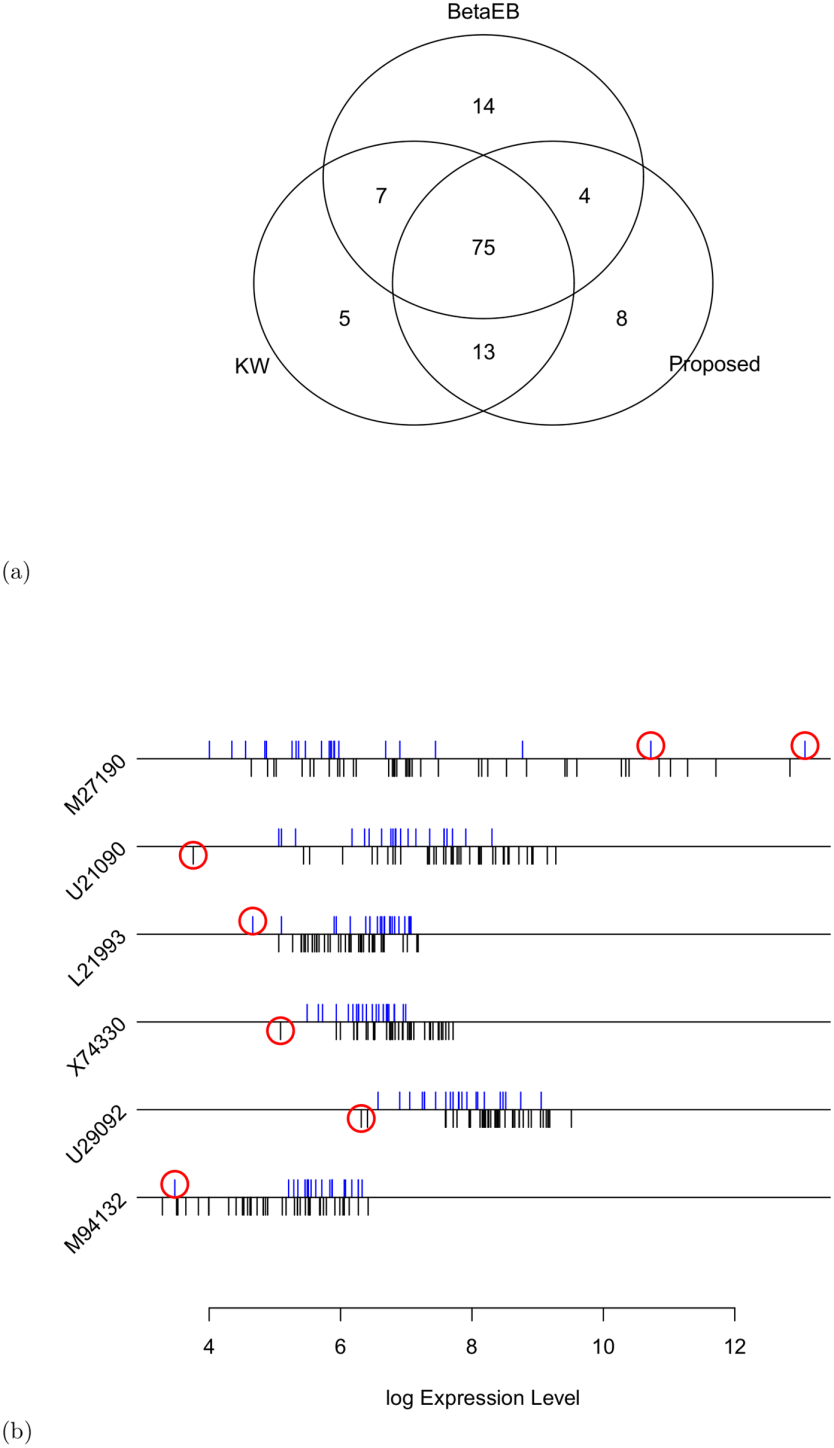
Venn diagram and outlier gene expression profile for colon cancer data. Comparison of the results on the colon cancer gene expression dataset. (a) Venn diagram of the top 100 genes estimated by KW, BetaEB and the proposed method. (b) Outlying DE genes detected by the proposed method only. The results for the control group are plotted below the lines, and the results for the cancer group are plotted above the lines.

This dataset has been analyzed in a previous study [28] and consists of 22 control and 40 colon cancer samples with 2000 genes. We downloaded this dataset from the website http://microarray.princeton.edu/oncology/affydata/index.html. We then directly applied three methods (KW, BetaEB and the proposed method) to this dataset, as before, to identify the DE genes. Fig 2a presents the Venn diagram of the top 100 DE genes estimated by each of the three methods (KW, BetaEB and Proposed). From this Venn diagram, it is evident that 75 DE genes are common to all three methods. The *β*-weight function of the proposed method identified 28% genes in the entire dataset as outliers. The outlier genes are indicated in red in S5(b)–S5(d) Fig. The proposed method identified 8 DE genes that were not detected by the other methods, of which five genes (UBE2I, R60883, PRIM1, POLD2 and REG1A) were upregulated and four genes (MUC2, ADCY2 and GLUT4) were downregulated. Six of these 8 genes were corrupted by outliers; these genes are indicated in Fig 2b by a red circle. To obtain some insights into the possible mechanisms that may be important in the development of resistance to drugs, the WebGestalt2 software package (available from http://bioinfo.vanderbilt.edu/webgestalt) [30] was used to query two pathway databases, KEGG (Kyoto Encyclopedia of Genes and Genomes) and GO (Gene Ontology) Pathways, for these 8 DE genes, yielding many important pathways to be enriched. We identified three important genes, which were described as L21993 (ADCY2; adenylate cyclase 2 (brain)), U21090 (POLD2; polymerase (DNA directed) delta 2, accessory subunit) and X74330 (PRIM1; primase DNA, polypeptide 1 (49 kDa)), which are involved in purine metabolism, DNA replication, pyrimidine metabolism and other metabolic pathways.

Using the GO database, we determined that POLD2 and PRIM1 are involved in biological processes and that the genes POLD2, PRIM1, U29092 (ubiquitin-conjugating enzyme E2I (UBE2I)), ADCY2, M94132 (human mucin 2 (MUC2) mRNA sequence), and M27190 (regenerating islet-derived 1 alpha (REG1A)) are involved in molecular functions and cellular components (S6 Fig We also investigated these 8 DE genes in the Oncomine database (http://www.oncomine.org). As summarized in Table C in S1 File, various independent studies have reported significant gene under-/over-expression in colon cancer tissues compared with corresponding normal tissues. We found that four independent studies have reported significant over-expression of PRIM1 (*p*-value ≤ 0.0002) in colon cancer tissues compared with corresponding normal tissues. Moreover, in one of these four studies, the rank of PRIM1 over-expression was within the top 4%, whereas in the remaining studies, this over-expression was ranked in the top 6-21%, similar to other genes (Table C in S1 File). These studies strongly suggest that these 8 genes may also be associated with colon cancer.

In our analysis, we also found some DE genes detected by other methods, such as only 5 genes by KW and 10 genes by BetaEB (Fig 2a). We applied all of these 15 genes to WebGestalt2 software for KEGG pathway analysis. Only five genes, X53743 (FBLN1: fibulin 1), X67699 (CD52: CD52 molecule), D38551 (RAD21: RAD21 homolog (S. pombe)), X16356 (CEACAM1: carcinoembryonic antigen-related cell adhesion molecule 1 (biliary glycoprotein)) and X07290 (ZNF3: zinc finger protein 3), were mapped using the KEGG Map in WebGestalt. We did not obtain any specific pathway for these genes, but some genes were very crucial from the previous investigations by different authors. As an example, the gene FBLN1 (fibulin 1) (detected by the KW approach only) was identified as a tumor suppressor gene whose inactivation may contribute to carcinogenesis [31]. It plays a tumor suppressive role in colorectal cancer (CRC)[32] and gastric cancer [33], among others. The gene RAD21 (detected only by the BetaEB method) is a component of the cohesion complex and is integral to chromosome segregation and error-free DNA repair. This gene expression in CRC is associated with aggressive disease, particularly in KRAS mutant tumors, and resistance to chemoradiotherapy. The gene RAD21 may be an important novel therapeutic target [34]. The gene CEACAM1 (detected only by the BetaEB method) is a tumor suppressor whose expression is known to be lost in the great majority of early adenomas and carcinomas. The loss of CEACAM1 expression is more common in neoplastic tumors than the adenomatous polyposis coli (APC) mutations [35]. Notably, these genes were also statistically significant by the proposed method with *p*-values (*p*) of 0.0003 < *p* < 0.0020, but they were not included in the top 100 genes. On the other hand, 8 DE genes of the proposed method were found by the KW and BetaEB approaches with *p*-values of 0.004 < *p* < 0.02 and posterior probabilities (*pp*) of 0.50 < *pp* < 1, respectively.

#### 3.2.3 Analysis of the Pancreatic Cancer Gene Expression Dataset with *m* = 4 Conditions

**Fig 3:**
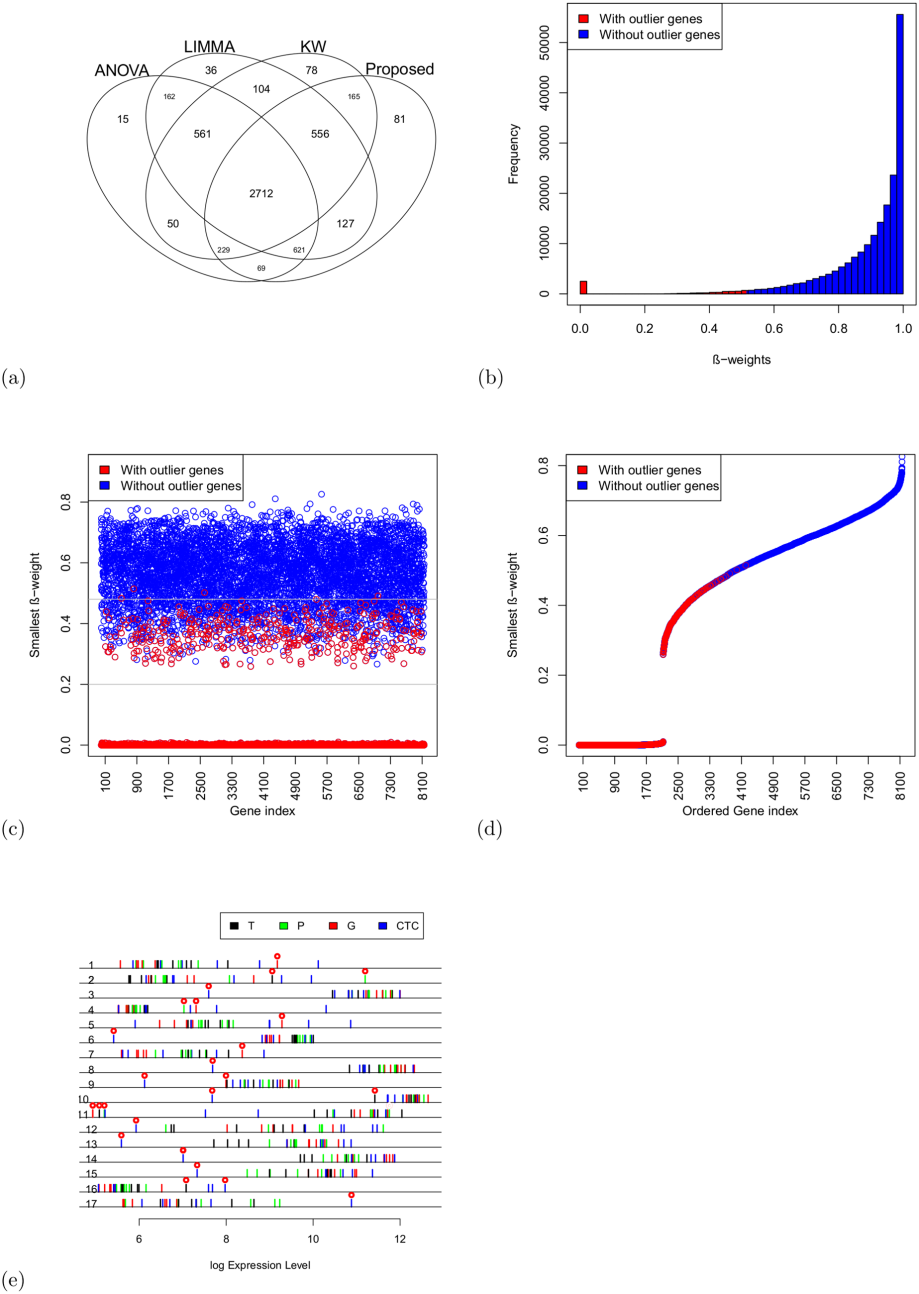
Venn diagram and outlier gene expression profile for pancreatic cancer data. (a) Venn diagram of the DE genes estimated by all four methods (ANOVA, LIMMA, KW and Proposed) based on pairwise comparisons of CTC vs T, CTC vs P, CTC vs G, T vs P, T vs G and G vs P. (b) Frequency distributions of *β*-weights for each expression of the 8152 genes in 24 samples. (c) Scatter plot of the smallest *β*-weight for each of the 8152 genes vs. the gene index, where the smallest value represents the minimum value of 24 *β*-weights from 24 samples for each gene. The red circles between the two gray lines represent moderate/noisy outliers, whereas the other red circles, corresponding to *β*-weights of less than 0.2, represent extreme outliers. (d) Plot of ordered smallest *β*-weights in (c) for 8152 genes. (e) The 80 DE genes detected by the proposed method only, as shown in (a). Seventeen out of 80 DE genes were detected as extreme outlying genes using the *β*-weight function. The results for the T, P, G and CTC groups are plotted above the lines with four different colors. The outlying samples are indicated by circles above them.

This dataset was used in the research reported in [29]. It consists of 24 samples, with 6 replicates in each of *m* = 4 conditions —circulation tumor samples (CTC), hematological cells (G), original tumor tissue (T), and non-tumor pancreatic control tissue (P) from patients and represents 8152 genes. We downloaded this dataset from the website http://www.ncbi.nlm.nih.gov/geo/query/acc.cgi?token=zbotduky//wssgujm/&acc=GSE18670. This dataset had been pre-processed by previous users [29]. Therefore, we directly applied four methods (ANOVA, LIMMA, KW and Proposed) to this dataset to test the null hypothesis (*H*_0_) of no differential expressions among four groups for each gene. If the *H*_0_ was rejected, we performed a pairwise comparison test for all 4 methods to identify the pattern of gene expressions. Fig 3a presents a Venn diagram of the DE genes estimated by the four methods based on pairwise comparisons of CTC vs T, CTC vs P, CTC vs G, T vs P, T vs G and G vs P, which was also the approach used in [29]. We used the Bonferroni method to adjust the *p*-values for ANOVA and KW and the Benjamini-Hochberg (BH) method to adjust the *p*-values for LIMMA and the proposed method. We used adjusted *p*-values at the 5% level of significance with an absolute fold change of 2 for each pairwise comparison in identifying DE genes. From this Venn diagram, it is evident that 2712 DE genes were common to all four methods in these 6 pairwise comparisons. The *β*-weight function of the proposed method identified 31% genes in the entire dataset as outliers. The outlier genes are indicated in red in Fig 3(b-d). However, there exist some outliers that may not influence the classical methods. In our simulation studies, we considered some special types of outliers that influence the classical methods. For example, let us consider a true EE gene between two groups as EE = {(20, 21, 22), (21, 20, 22)} and a true DE gene between two groups as DE = {(20, 21, 22), (26, 27, 28)}, where () is used to represent the samples of expressions from a group. In the case of an EE gene, we observe that the absolute mean difference between two groups is 0, whereas in the case of a DE gene, the absolute mean difference between two groups is 6. If we corrupted the EE gene by an outlier expression as EE1 = {(20, 59*, 22), (21, 60*, 22)}, EE2 = {(20, 0*, 22), (21, 1*, 22)}, EE3 = {(20, 59*, 22), (21, 90*, 22)}, EE4 = {(20, 21, 22), (21, 60*, 22)}, we will observe that outliers (*) in EE1 and EE2 cannot influence the classical/non-robust methods, whereas outliers (*) in EE3 and EE4 can highly influence the classical/non-robust methods. Similarly, if we corrupted the previous DE gene by an outlier expression as DE1 = {(20, 21, 22), (26, 70*, 28)}, DE2 = {(20, 1*, 22), (26, 27, 28)}, DE3 = {(20, 1*, 22), (26, 70*, 28)}, DE4 = {(20, 21, 22), (26, 8*, 28)}, DE5 = {(20, 38*, 22), (26, 27, 28)}, DE6 = {(20, 29*, 22), (26, 17*, 28)}, we will observe that the assumed outliers (*) in DE1- DE3 cannot influence the classical/non-robust methods, whereas outliers (*) in DE4-DE6 can highly influence the classical/non-robust methods. The group variance/scale is 1 in both cases of original EE/DE genes, whereas the group variance/scale lies between 1 and 1565 in both cases of outlying EE/DE genes. In the current real data analysis, we found that the proposed method detected 80 DE genes that were not detected by any of the other methods. Among these 80 DE genes, 35 genes were corrupted by outliers. Among these 35 outlying genes, we considered 17 extreme outlying genes expressions for functional analysis to investigate their importance as an example. These outlying genes were then plotted above the lines with four different colors for the T, P, G and CTC groups, where the outlier samples are indicated by red circles above (Fig 3e). To compare all possible comparisons, only genes with significant 2-fold up/down regulation were selected (using adjusted *p*-values ≤ 0.05) for all methods (Table 9). In this table, we observed a large number of DE genes detected by the proposed method. We searched for functional enrichment of specific pathways by these 17 DE genes using the WebGestalt gene analysis toolkit [30]. By mapping the differentially expressed gene set against the biological function annotations in the Gene Ontology database, we found significant enrichment of genes involved in the positive regulation of muscle-cell differentiation, the immune-response-regulating cell surface receptor signaling pathway, and the antigen-receptor-mediated signaling pathway, as well as genes involved in the carboxylic acid biosynthetic process (S2 File (xls)). Using the KEGG database, we found that several probes mapped to signaling pathways, including the beta-Alanine metabolism pathway, the MAPK signaling pathway and other metabolic pathways (Table 10 and S3 File (xls)). The pathway with the highest expression ratio in CTC was the p38 mitogen-activated protein kinase (p38 MAPK) signaling pathway, which is known to be involved in cancer cell migration. In the p38 MAPK pathway, PP2CB and MEF2C were significantly upregulated. The simulation study results in Table 7 also increase our confidence in the results of the proposed method for this real dataset because that simulated dataset was generated in accordance with the parametric properties of this real dataset.

**Table 9:** Pairwise comparison analysis by all 4 methods with their corresponding selected significance DE genes.

Numbers of DR and UR genes				
Corrected p-value < 0.05				
Pair of groups	Predicted DR ${\left(\text{log}\frac{{\hat{\mu }}_{i}}{{\hat{\mu }}_{j}}<-1\right)}^{a}$	Overlapped predicted DR	Predicted UR ${\left(\text{log}\frac{{\hat{\mu }}_{i}}{{\hat{\mu }}_{j}}>+1\right)}^{a}$	Overlapped predicted UR
CTC vs T	{1570, 1909, 1533, **1710**}	1367	{1392, 1663, 1294, **1590**}	1215
CTC vs P	{1589, 1904, 1590, **1720**}	1413	{1446, 1738, 1323, **1675**}	1247
CTC vs G	{612, 911, 463, **826**}	354	{744, 938, 618, **916**}	534
T vs P	{20, 0, 25, **60**}	0	{51, 7, 72, **94**}	7
T vs G	{1001, 1179, 1125, **1122**}	983	{890, 1132, 1107, **1070**}	856
P vs G	{1081, 1281, 1215, **1220**}	1048	{898, 1191, 1165, **1094**}	857

The values reported in the form {x, x, x, x} in this table represent the numbers of downregulated (DR) or upregulated (UR) differentially expressed (DE) genes estimated by the ANOVA, LIMMA, KW and proposed (Bold) methods, respectively. ^*a*^Note that $\text{log}\frac{{\hat{\mu }}_{i}}{{\hat{\mu }}_{j}}<-1$ indicates significant 2-fold downregulation and $\text{log}\frac{{\hat{\mu }}_{i}}{{\hat{\mu }}_{j}}>+1$ indicates significant 2-fold upregulation.

**Table 10:** KEGG pathways for the 17 DE genes identified by the proposed method only.

KEGG ID	KEGG pathway description	No. of genes (%)	*p*-values	Adjusted *p*-values
hsa00410	beta-Alanine metabolism	2 (11.76)	2.25e-05	6.75e-05
hsa04010	MAPK signaling pathway	2 (11.76)	0.0033	0.0099
hsa01100	metabolic pathways	2 (11.76)	0.0507	0.1521

KEGG terms that are significantly enriched in the 17 pancreatic-cancer-related genes identified by the proposed method only. The *p*-values were estimated using the hypergeometric test and then adjusted via the Bonferroni multiple testing correction (adjusted *p*-values).

## 4 Conclusion

We proposed a hybrid one-way ANOVA approach that unifies the robustness and efficiency of estimation of model parameters for the discovery of differential gene expressions with two or more conditions with multiple patterns. The proposed approach is controlled by the *β*-weight function of the minimum *β*-divergence method such that MLEs are used in the absence of outliers and minimum *β*-divergence estimators are used in the presence of outliers for estimating the group parameters in the ANOVA model. The proposed method produces robust and efficient results because MLEs are consistent and asymptotically efficient under a Gaussian distribution in the absence of outliers and because the minimum *β*-divergence estimators are highly robust and asymptotically efficient in the presence of outliers. It overcomes the problems that arise in the existing robust methods for both small- and large-sample cases with multiple patterns of gene expression. The *β*-weight function plays the key role in the performance of the proposed method. It can accurately and significantly detect outlying expressions.

The simulation results showed that all eight methods (ANOVA, SAM, LIMMA, EBarrays, eLNN, KW, robust BetaEB and proposed) perform almost identically for both the small- and large-sample cases with *m* = 2 conditions in the absence of outliers. The robust BetaEB method and the proposed method exhibited better performance than the other six methods in the presence of at most 50% outlying genes (although we provided results with 5%, 10% and 75% outlying genes only) with one or two outliers for each outlying gene. However, the BetaEB approach appears to be slightly better than the proposed method for the small-sample case, whereas the proposed method exhibited considerably better performance than the BetaEB method for both the small- and large-sample cases in the presence of more than 50% outlying genes with 5% outlier samples for each outlying gene. To investigate the performance of the proposed method in the case of multiple (*m* > 2) conditions with multiple patterns of expression, four popular methods (ANOVA, SAM, LIMMA and KW) were considered based on the availability of software/extended-version of the algorithms for testing the equality multiple means. The non-parametric approach KW exhibited weak performance in a comparison of four methods (ANOVA, SAM, LIMMA and proposed) for the small-sample case with *m* > 2 conditions in the absence of outliers. However, the proposed method exhibited improved performance compared to the other methods for the small-sample case in the presence of outliers. The non-parametric KW method and the proposed method showed better performance in a comparison of the other three methods (ANOVA, SAM and LIMMA) for the large-sample case with *m* > 2 conditions in the presence of few outlying genes, where the proposed method appears to be slightly better than the KW method. The results on real gene expression datasets demonstrated that the proposed method can identify some additional outlying genes as differential expression compared to the other methods. Moreover, we found that the identified genes are reported as being important genes in different studies by other researchers. Therefore, the proposed approach would be more suitable and reliable on average for the identification of DE genes between two or more conditions with multiple patterns of expression.

## Supporting Information

S1 Fig Plot of FDR versus number of top DE genes estimated by different methods. (a) In the absence of outlying genes. (b) In the presence of one outlying expression in 5% genes. (c) In the presence of one outlying expression in 10% genes. (d) In the presence of one outlying expression in 75% genes.
(TIF)

S2 Fig Plot of FNR versus FPR estimated by different methods. (a) In the absence of outlying genes. (b) In the presence of one outlying expression in 5% genes. (c) In the presence of one outlying expression in 10% genes. (d) In the presence of one outlying expression in 75% genes.
(TIF)

S3 Fig ROC curves produced by different methods. (a) In the absence of outlying genes. (b) In the presence of one outlying expression in 5% genes. (c) In the presence of one outlying expression in 10% genes. (d) In the presence of one outlying expression in 75% genes.
(TIF)

S4 Fig Results of spike data analysis. (a) Frequency distribution of *β*-weights for each expression of 18707 genes with 18 samples. (b) Scatter plot of the smallest *β*-weight for each of the 18707 genes vs. the gene index, where the smallest value represents the minimum value of 18 *β*-weights from 18 samples for each gene. The red circles between the two gray lines represent moderate/noisy outliers, whereas the remaining red circles, corresponding to *β*-weights of less than 0.2, represent extreme outliers. (c) Ordered plot of the smallest *β*-weights shown in (b) for the 18707 genes. (d) Bar plots based on the outlying designated EE genes detected by the proposed *β*-weight function. (e) Bar plots based on the outlying designated DE genes detected by the proposed *β*-weight function. (f) M-A plot based on the group medians, where red stars (⋆) are used for the 233 outlying designated EE genes detected by the proposed method with *p*–*value* < 0.05 shown in (d), and blue stars (⋆) are used for the 40 outlying designated DE genes detected by the proposed method with *p*–*value* < 0.05 shown in (e).
(TIFF)

S5 Fig Results of colon cancer data analysis. (a) Venn diagram of the top 100 genes estimated by BetaEB, KW and the proposed methods. (b) Frequency distribution of *β*-weights for each expression of 2000 genes with 61 samples. (c) Scatter plot of the smallest *β*-weight for each of the 2000 genes vs. the gene index, where the smallest value represents the minimum value of 61 *β*-weights from 61 samples for each gene. The red circles between the two gray lines represent moderate/noisy outliers, whereas the remaining red circles, corresponding to *β*-weights of less than 0.2, represent extreme outliers. (d) Ordered plot of the smallest *β*-weights shown in (c) for the 2000 genes.
(TIF)

S6 Fig Gene ontology (GO) categories of eight (8) genes. This directed acyclic graph (DAG) shows the gene ontology categories of eight (8) genes, detected by the proposed method only in the colon cancer dataset, obtained using the WebGestalt database. These enriched GO categories were hierarchically organized into a DAG tree; each box in the tree lists the name of the GO category, the number of genes in that category, and the FDR-adjusted *p*-value (adjP) if the enrichment is significant. The categories shown in red are enriched (adjusted *p*-value of < 0.05), whereas those in black are non-enriched.
(TIF)

S1 File Results for simulated and real gene expression datasets. Performance evaluations based on simulated gene expression profiles are presented in Tables A and B and real gene expression colon cancer dataset results are presented in Table C.
(PDF)

S2 File GO Pathways for 17 pancreatic cancer DE genes. Gene Ontology analysis information.
(XLS)

S3 File KEGG Pathways for 17 pancreatic cancer DE genes. KEGG analysis information.
(XLS)
