# Supplementary figures and images for: A Hybrid One-Way ANOVA Approach for the Robust and Efficient Estimation of Differential Gene Expression with Multiple Patterns
Source: PLoS One. 2015 Sep 28;10(9):e0138810. doi: 10.1371/journal.pone.0138810 (PMC4587675; doi:10.1371/journal.pone.0138810)

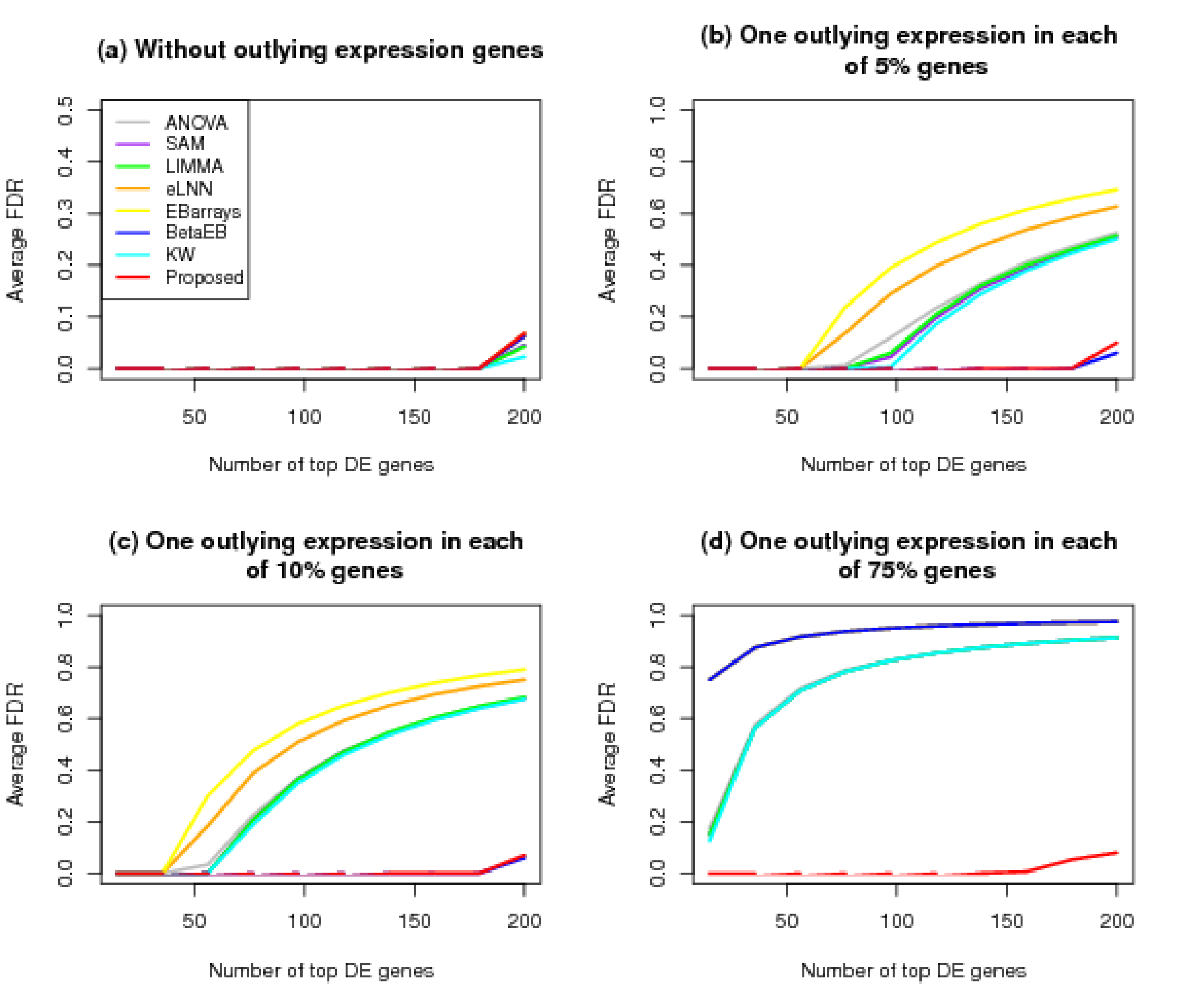

Supplement: S1 Fig — (a) In the absence of outlying genes. (b) In the presence of one outlying expression in 5% genes. (c) In the presence of one outlying expression in 10% genes. (d) In the presence of one outlying expression in 75% genes. (TIF) [file pone.0138810.s001.tif]

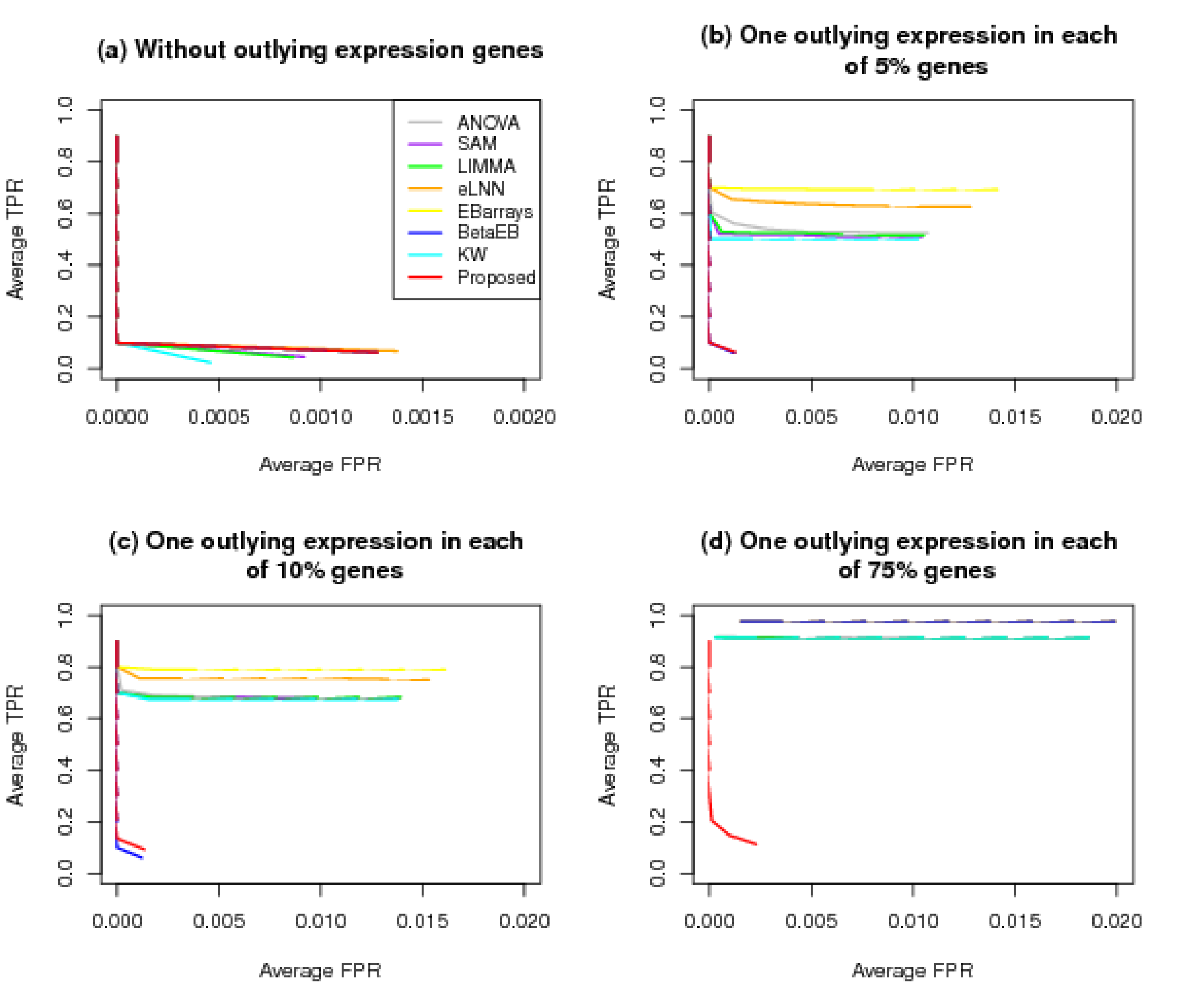

Supplement: S2 Fig — (a) In the absence of outlying genes. (b) In the presence of one outlying expression in 5% genes. (c) In the presence of one outlying expression in 10% genes. (d) In the presence of one outlying expression in 75% genes. (TIF) [file pone.0138810.s002.tif]

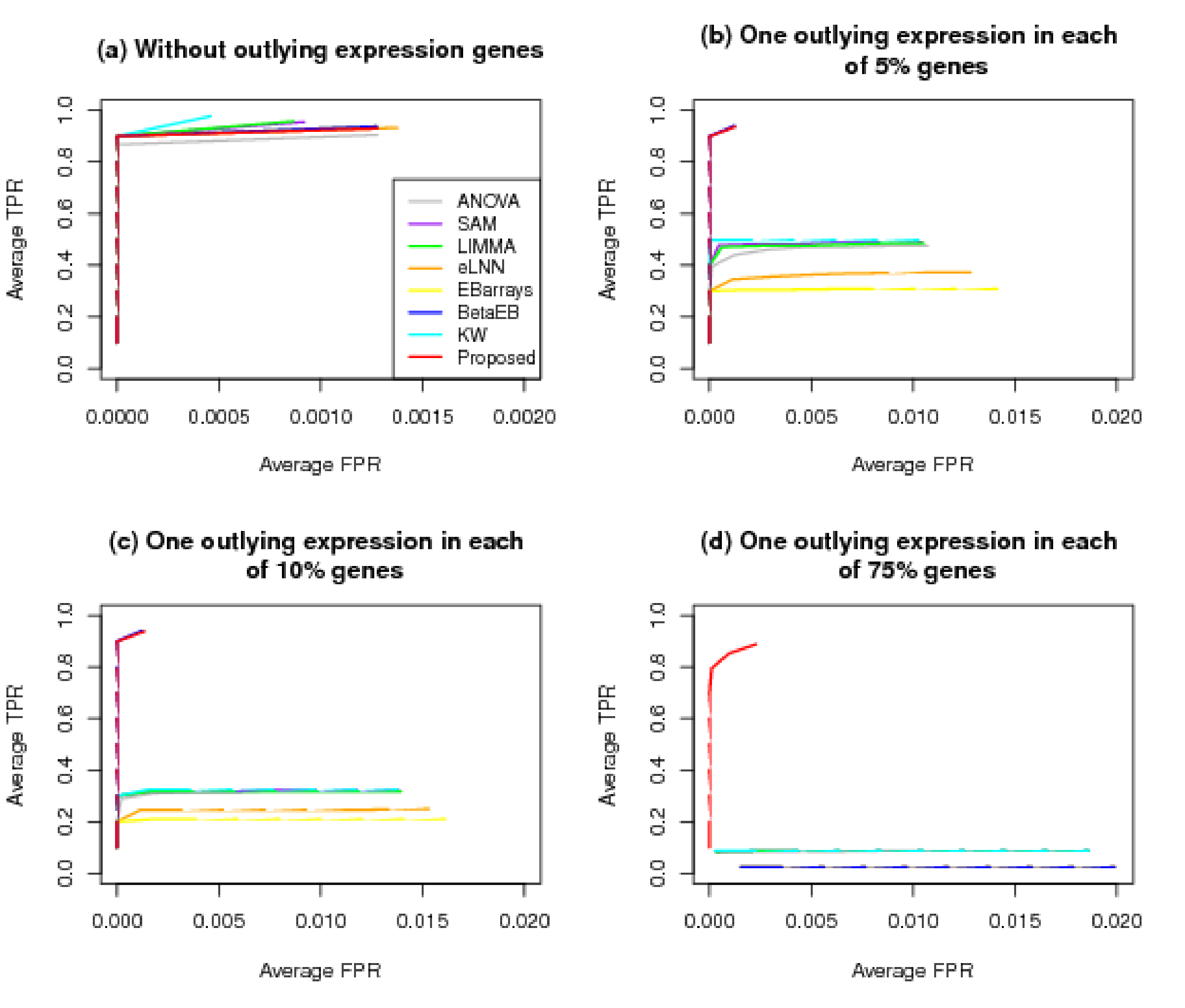

Supplement: S3 Fig — (a) In the absence of outlying genes. (b) In the presence of one outlying expression in 5% genes. (c) In the presence of one outlying expression in 10% genes. (d) In the presence of one outlying expression in 75% genes. (TIF) [file pone.0138810.s003.tif]

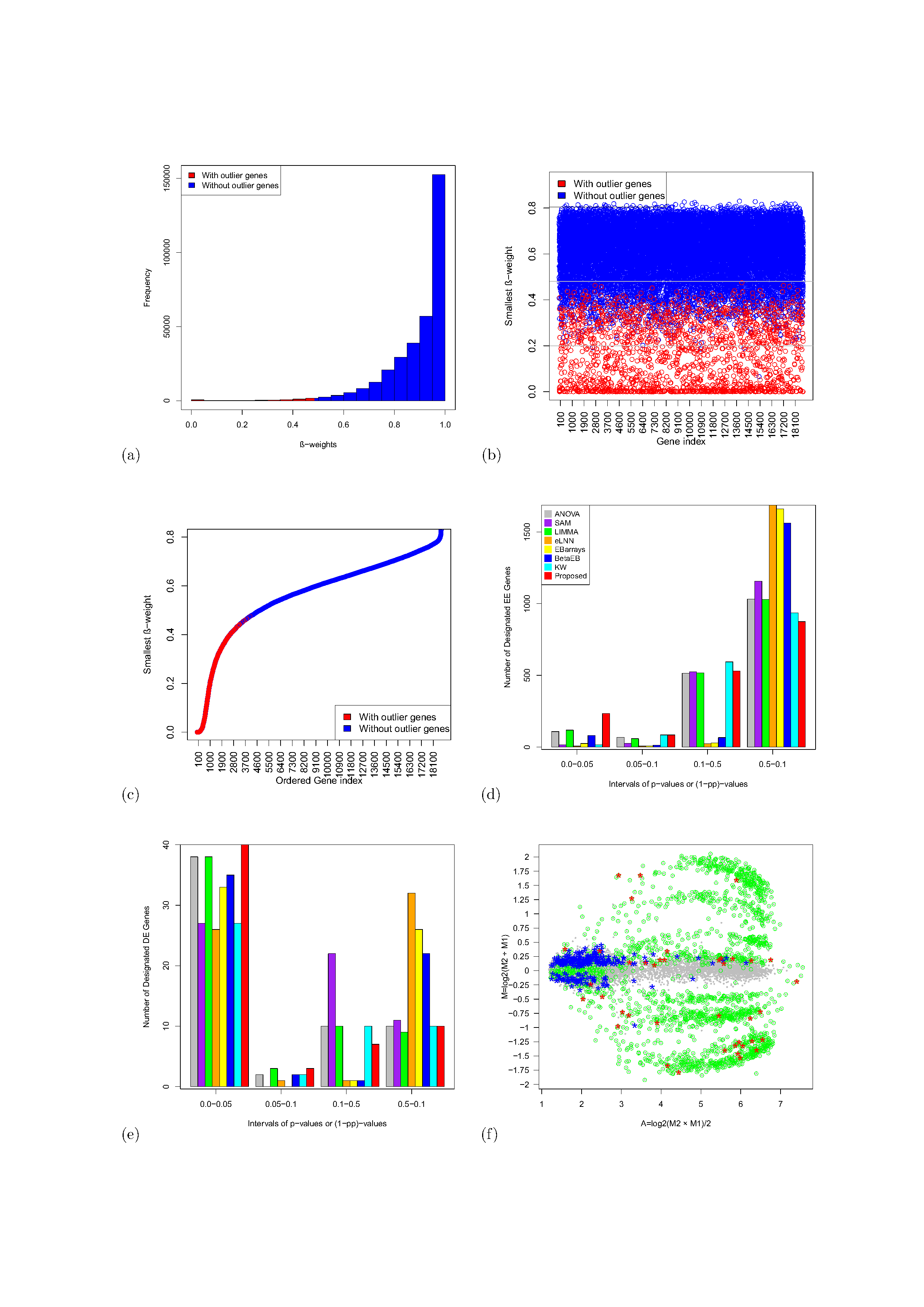

Supplement: S4 Fig — (a) Frequency distribution of β-weights for each expression of 18707 genes with 18 samples. (b) Scatter plot of the smallest β-weight for each of the 18707 genes vs. the gene index, where the smallest value represents the minimum value of 18 β-weights from 18 samples for each gene. The red circles between the two gray lines represent moderate/noisy outliers, whereas the remaining red circles, corresponding to β-weights of less than 0.2, represent extreme outliers. (c) Ordered plot of the smallest β-weights shown in (b) for the 18707 genes. (d) Bar plots based on the outlying designated EE genes detected by the proposed β-weight function. (e) Bar plots based on the outlying designated DE genes detected by the proposed β-weight function. (f) M-A plot based on the group medians, where red stars (⋆) are used for the 233 outlying designated EE genes detected by the proposed method with p–value < 0.05 shown in (d), and blue stars (⋆) are used for the 40 outlying designated DE genes detected by the proposed method with p–value < 0.05 shown in (e). (TIFF) [file pone.0138810.s004.tiff]

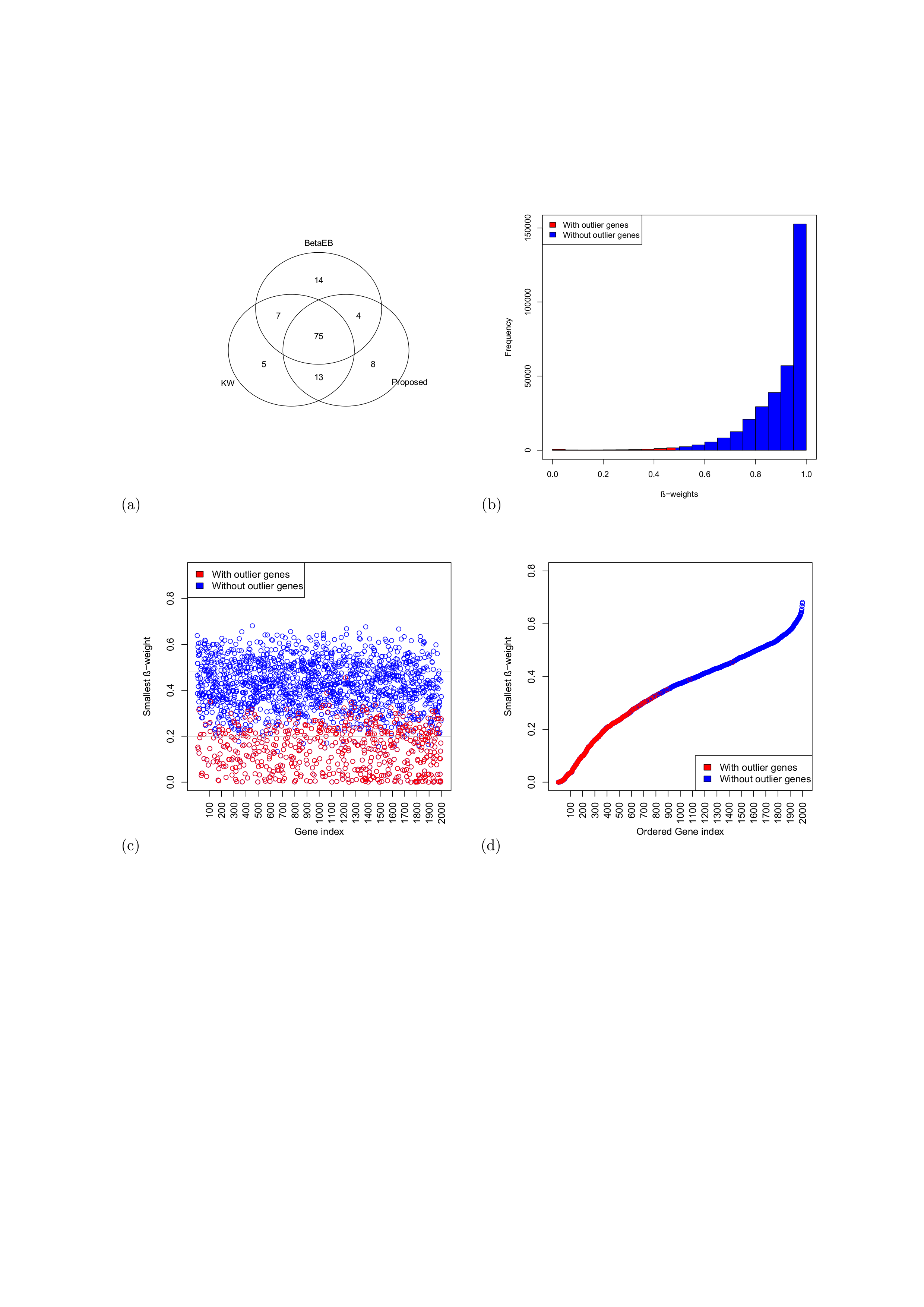

Supplement: S5 Fig — (a) Venn diagram of the top 100 genes estimated by BetaEB, KW and the proposed methods. (b) Frequency distribution of β-weights for each expression of 2000 genes with 61 samples. (c) Scatter plot of the smallest β-weight for each of the 2000 genes vs. the gene index, where the smallest value represents the minimum value of 61 β-weights from 61 samples for each gene. The red circles between the two gray lines represent moderate/noisy outliers, whereas the remaining red circles, corresponding to β-weights of less than 0.2, represent extreme outliers. (d) Ordered plot of the smallest β-weights shown in (c) for the 2000 genes. (TIF) [file pone.0138810.s005.tif]

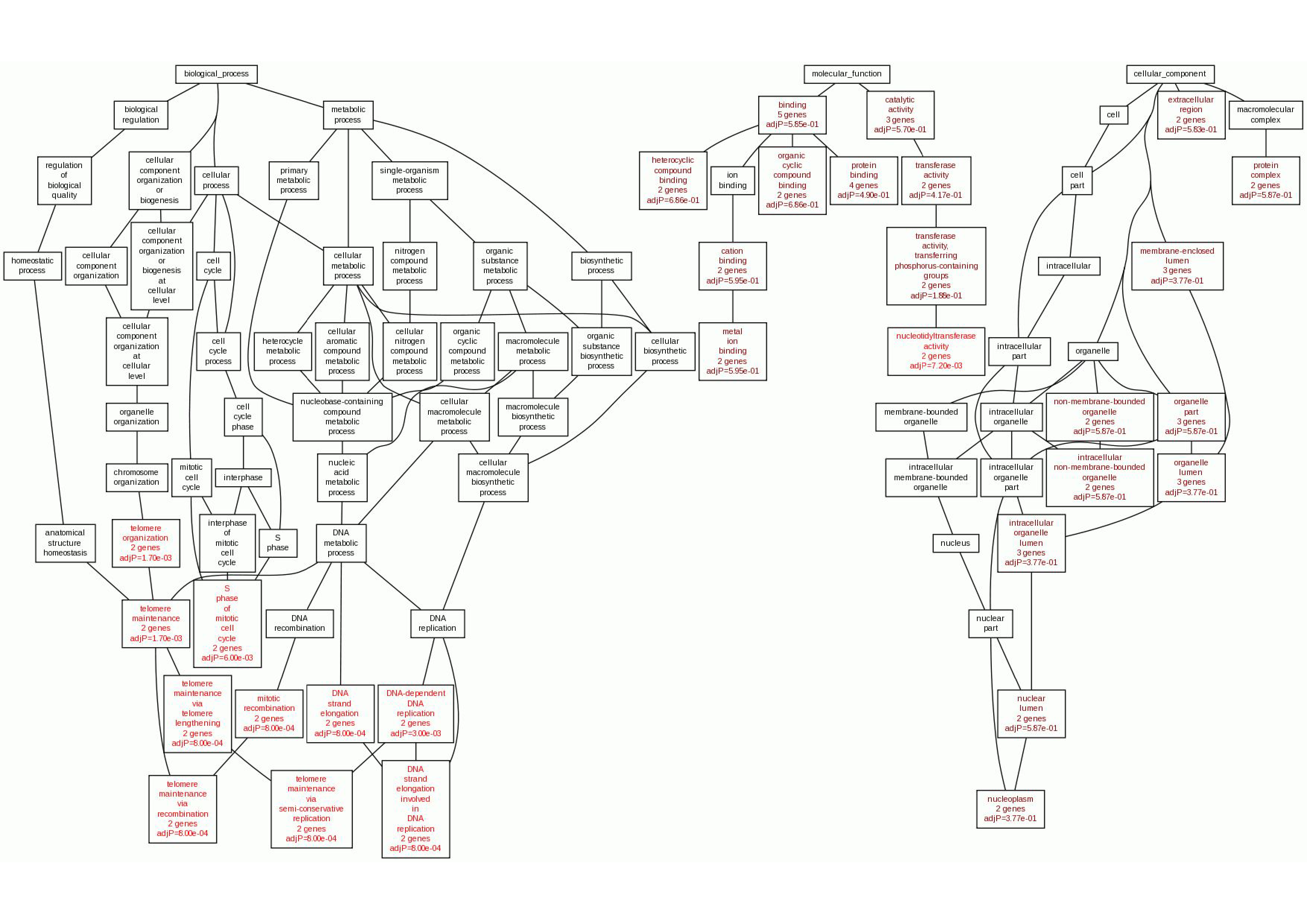

Supplement: S6 Fig — This directed acyclic graph (DAG) shows the gene ontology categories of eight (8) genes, detected by the proposed method only in the colon cancer dataset, obtained using the WebGestalt database. These enriched GO categories were hierarchically organized into a DAG tree; each box in the tree lists the name of the GO category, the number of genes in that category, and the FDR-adjusted p-value (adjP) if the enrichment is significant. The categories shown in red are enriched (adjusted p-value of < 0.05), whereas those in black are non-enriched. (TIF) [file pone.0138810.s006.tif]
