# Supplementary material for: A Hybrid One-Way ANOVA Approach for the Robust and Efficient Estimation of Differential Gene Expression with Multiple Patterns
Source: PLoS One. 2015 Sep 28;10(9):e0138810. doi: 10.1371/journal.pone.0138810 (PMC4587675; doi:10.1371/journal.pone.0138810)
Supplement: S1 File — Performance evaluations based on simulated gene expression profiles are presented in Tables A and B and real gene expression colon cancer dataset results are presented in Table C. (PDF) [file pone.0138810.s007.pdf]

A Hybrid One-Way ANOVA Approach for Robust and Efficient Estimation of Differential Gene-Expressions with Multiple Patterns

Mohammad Manir Hossain Mollah[1,*], Rahman Jamal[1], Norfilza Mohd Mokhtar[1,2] Roslan Harun[1], Md. Nurul Haque Mollah[3]

1 Institut Perubatan Molekul UKM (UMBI), University Kebangsaan Malaysia (UKM), Jalan Ya'acob Latiff, Bandar Tun Razak, Cheras 56000 Kuala Lumpur, Malaysia

2 Department of Physiology, Faculty of Medicine, Universiti Kebangsaan Malaysia, Kuala Lumpur, Malaysia

3 Laboratory of Bioinformatics, Department of Statistics, University of Rajshahi, Rajshahi-6205, Bangladesh.

∗ E-mail: mhmollah06@yahoo.com

1. Hybridization of Robustness and Efficiency of Estimation in One-Way ANOVA Using the Minimum β-Divergence Method

Let x_{jk} be the kth observed random expression of a gene in the jth condition $(j = 1, 2, ..., m; k = 1, 2, ..., n_j)$, which follows the one-way ANOVA model as expressed below:

$$x_{jk} = \mu_j + \epsilon_{jk}, \tag{1}$$

where μ_j is the mean of all expressions of a gene in the jth condition and ϵ_{jk} is the random error term that follows $N(0, \sigma_j^2)$. We wish to test the null hypothesis $(H_0) : \mu_1 = \mu_2 = ... = \mu_m = \mu$ against the alternative hypothesis $(H_1) : H_0$ is not true, assuming that $\sigma_1^2 = \sigma_2^2 = ... = \sigma_m^2 = \sigma^2$. Thus, the generalized likelihood-ratio test (LRT) criterion yields the following F-statistic to test H_0 against H_1:

$$\begin{aligned} F &= \frac{\sum_{j=1}^{m} n_j(\hat{\mu}_j - \hat{\mu})^2/(m-1)}{\sum_{j=1}^{m}\sum_{k=1}^{n_j}(\boldsymbol{x}_{jk} - \hat{\mu}_j)^2/(n-m)} \\ &= \frac{\sum_{j=1}^{m} n_j(\hat{\mu}_j - \hat{\mu})^2/(m-1)}{[n_1\hat{\sigma}_1^2 + n_2\hat{\sigma}_2^2 + ... + n_m\hat{\sigma}_m^2]/(n-m)}, \end{aligned} \tag{2}$$

which follows the F-distribution with (m-1) and (n-m) degrees of freedom under H_0 [1], where $n = n_1 + n_2 +, ..., +n_m$ and

$$\hat{\mu}_j = \frac{1}{n_j}\sum_{k=1}^{n_j} x_{jk}, \tag{3}$$

$$\hat{\sigma}_j^2 = \frac{1}{n_j}\sum_{k=1}^{n_j}(x_{jk} - \hat{\mu}_j)^2, \tag{4}$$

$$\hat{\mu} = \frac{1}{n}\sum_{j=1}^{m}\sum_{k=1}^{n_j} x_{jk} = \sum_{j=1}^{m} n_j\hat{\mu}_j/n. \tag{5}$$

The critical region (CR) for testing H_0 against H_1 at the (1-α)100% level of significance is defined by $Pr[F \geq F_0|H_0] = \alpha$, where $F_0 = F_\alpha(m-1, n-m)$ is the upper 100α% points of the F-distribution with $m-1$ and $n-m$ degrees of freedom. F_0 is also known as the cut-off point or critical value of the test. However, it is obvious that the maximum likelihood estimates (MLEs) $\widehat{\boldsymbol{\theta}}_j = (\hat{\mu_j}, \hat{\sigma}_j^2)$ of $\theta_j = (\mu_j, \sigma_j^2)$ for $j = 1, 2, ..., m$ in the above equations 3 and 5 are highly sensitive to

outliers. Therefore, the identification of DE genes using classical ANOVA may produce misleading results because gene expression data are often contains outliers. Thus, in this paper, we consider the minimum β-divergence method [2, 3] to improve the robustness and efficiency of estimation in one-way ANOVA. The minimum β-divergence estimators $\widehat{\boldsymbol{\theta}}_{j,\beta} = (\hat{\mu}_{j,\beta}, \hat{\sigma}^2_{j,\beta})$ of the parameters $\boldsymbol{\theta}_j = (\mu_j, \sigma_j^2)$ are computed iteratively as follows:

$$\mu_{j,t+1} = \frac{\sum_{k=1}^{n_j} \phi_\beta(x_{jk}|\boldsymbol{\theta}_{j,t}) x_{jk}}{\sum_{k=1}^{n_j} \phi_\beta(x_{jk}|\boldsymbol{\theta}_{j,t})} \tag{6}$$

and

$$\sigma^2_{j,t+1} = \frac{\sum_{k=1}^{n_j} \phi_\beta(x_{jk}|\boldsymbol{\theta}_{j,t})(x_{jk} - \mu_{j,t})^2}{(\beta+1)^{-1} \sum_{k=1}^{n_j} \phi_\beta(x_{jk}|\boldsymbol{\theta}_{j,t})}, \tag{7}$$

where

$$W_\beta(x_{jk}|\boldsymbol{\theta}_j) = \exp\{-\frac{\beta}{2\sigma_j^2}(x_{jk} - \mu_j)^2\}, \tag{8}$$

which we call the β-weight function [2, 3]. The notation $\boldsymbol{\theta}_{t+1}$ represents the update to $\boldsymbol{\theta}_t$ in the $(t+1)$th iteration. The robustness of these estimators is discussed in the context of influence functions in [2], and their consistency is discussed in [3]. The minimum β-divergence estimators $\widehat{\boldsymbol{\theta}}_{j,\beta} = (\hat{\mu}_{j,\beta}, \hat{\sigma}^2_{j,\beta})$ of the parameters $\boldsymbol{\theta}_j = (\mu_j, \sigma_j^2)$ can tolerate up to 50% outlying expressions/observations in the dataset if appropriate initial values are chosen for $\boldsymbol{\theta}_j$ in equations 6 and 8). To obtain these appropriate initial values, we define a dataset $\mathscr{D}_j^0 \subset \mathscr{D}_j = \{x_{jk};\ k = 1, 2, ..., n_j\}$ as follows:

$$\mathscr{D}_j^0 = \{x_{jk} \in \mathscr{D}_j;\ |x_{jk} - x_j^0| < x_j^1, k = 1, 2, ..., n_j\}, \tag{9}$$

where x_j^0 and x_j^1 are the medians of X_j and $|X_j - x_j^0|$, respectively. Then, the appropriate initial value for $\boldsymbol{\theta}_j = (\mu_j, \sigma_j^2)$ is computed using the classical estimators (equations 3 and 4) based on the sub-dataset $\mathscr{D}_j^0$. For β=0, the minimum β-divergence estimators $\widehat{\boldsymbol{\theta}}_{j,\beta}$ (equations 6 and 7) reduce to the non-iterative MLEs $\widehat{\boldsymbol{\theta}}_j$ (equations 3 and 4).

It is well known that MLE is more efficient than any robust estimator in the absence of outliers. Therefore, in this paper, an attempt is made to develop a hybrid approach in which the classical estimators $\widehat{\boldsymbol{\theta}}_j$ (equations 3 and 4) are used in the absence of outliers and the minimum β-divergence estimators $\widehat{\boldsymbol{\theta}}_{j,\beta}$ (equations 6 and 7) are used in the presence of outliers for the estimation of $\boldsymbol{\theta}_j$ in one-way ANOVA. The minimum β-divergence method offers two approaches to unifying robustness and efficiency of estimation in ANOVA. One method is to select the tuning parameter β via cross-validation (CV), as discussed in detail in a previous publication [2]. In the absence of outliers, the CV method produces β=0 for the minimum β-divergence estimators and is thus equivalent to the classical estimators, as discussed above. In the presence of outliers, it produces $\beta > 0$ for the minimum β-divergence estimators. To develop the alternative approach, we consider the β-weight function (equation 8) with $\beta = 0.2$ for outlier detection. This weight function assigns smaller weights (≥ 0) to outlier observations and larger weights (≤ 1) for no outlying observations. A gene expression x_{jk} is defined based on the β-weight function as follows, depending on whether it is contain outlier or not:

$$W_\beta(x_{jk}|\widehat{\boldsymbol{\theta}}_{j,\beta}) = \begin{cases} > \delta_j, \text{ if } x_{jk} \text{ is not outlying} \\ \leq \delta_j, \text{ if } x_{jk} \text{ is outlying,} \end{cases} \tag{10}$$

where the threshold value δ_j is the quantile value of $W_\beta(x_{jk}|\widehat{\boldsymbol{\theta}}_{j,\beta})$ with probability

$$Pr\{W_\beta(x_{jk}|\widehat{\boldsymbol{\theta}}_{j,\beta}) \leq \delta_j\} \leq p = 10^{-5}. \tag{11}$$

Here, the derivation of the distributional form of $W_\beta(x_{jk}|\widehat{\boldsymbol{\theta}}_{j,\beta})$ is not a tractable problem. However, if we assume that $\widehat{\boldsymbol{\theta}}_{j,\beta} = \boldsymbol{\theta}_j$ (good fit), then we can consider the distribution

$$W_\beta(x_{jk}|\widehat{\boldsymbol{\theta}}_{j,\beta}) \rightsquigarrow \frac{2}{\beta \times \delta_j} f_{\chi^2_{(1)}}(-\frac{2}{\beta}\log\delta_j), \tag{12}$$

where $\chi^2_{(1)}$ denotes the chi-square variable with 1 degree of freedom, which assumes values of $-\frac{2}{\beta}\log\delta_j$, where $0 < \delta_j \leq 1$. We can also simulate the distribution of $W_\beta(y_j|\widehat{\boldsymbol{\theta}}_{j,\beta})$ to obtain the threshold value δ_j, where the values of the y_js are simulated based on the normal distribution with mean $\hat{\mu}_{j,\beta}$ and variance $\hat{\sigma}^2_{j,\beta}$. Thus, we can unify the minimum β-divergence estimator with MLE for $\boldsymbol{\theta}_j$ in the jth condition as follows:

$$\widehat{\boldsymbol{\theta}}_{j,\beta} = \begin{cases} \widehat{\boldsymbol{\theta}}_{j,\beta}, & \text{if } \sum_{k=1}^{n_j} I_{[W_\beta(x_{jk}|\widehat{\boldsymbol{\theta}}_{j,\beta})>\delta_j]} < n_j, \\ \widehat{\boldsymbol{\theta}}_j, & \text{if } \sum_{k=1}^{n_j} I_{[W_\beta(x_{jk}|\widehat{\boldsymbol{\theta}}_{j,\beta})>\delta_j]} = n_j. \end{cases}$$

Then, the modified F-statistic, denoted by F_β, is given by

$$F_\beta = \frac{\sum_{j=1}^{k} n_j(\hat{\mu}_{j,\beta} - \hat{\mu}_\beta)^2/(k-1)}{[n_1\hat{\sigma}^2_{1,\beta} + n_2\hat{\sigma}^2_{2,\beta} + ... + n_m\hat{\sigma}^2_{m,\beta}]/(n-m)}. \tag{13}$$

To test the null hypothesis (H_0) against the alternative hypothesis (H_1) from the robustness perspective, we can compute p-values under the assumption that F_β approximately follows the F-distribution. Note that this modified F-statistic (F_β) reduces to the classical F-statistic (equation 2) for $\beta = 0$. However, we can also compute permutation-based p-values to test whether H_0 is true or false. To compute permutation-based p-values, we first compute the value of F_β as defined by equation 13 based on the given dataset. Then, we permute the values of the given dataset of all conditions N times, and each time, we compute F_β. Finally, we compute the p-values for testing H_0 against H_1 using the following formula:

$$p\text{-value} = \sum_{k=1}^{N} I_{[\hat{F}_\beta(k) \leq \hat{F}_\beta]}/N, \tag{14}$$

where $\hat{F}_\beta$ denotes the estimate of F_β obtained for the given dataset and $\hat{F}_\beta(k)$ denotes the estimate of F_β obtained for the kth permutation of the values of the response variable in the dataset. Note that for β=0, F_β reduces to the classical F-statistic.

2. Supplementary Results for simulated and Real Gene-Expressions Datasets

Performance investigation for the proposed method against other eight several methods based on simulated gene-expression profiles under m=2 conditions in **subsection 2.1**. Supplementary results of the proposed method for the real gene expression colon cancer dataset is given in the following **subsection 2.2**. Discussions about these supplementary results are given in the main text.

2.1. Performance evaluation based on simulated gene expression profiles with m=2 conditions for both small- and large-sample cases

Table A: Performance evaluation based on simulated gene expression profiles with m=2 conditions for the small-sample case ($\mathbf{n_1 = n_2 = 4}$)

	Results for the small-sample case ($\mathbf{n_1 = n_2 = 4}$)															
Methods	TPR	FPR	TNR	FNR	FDR	MER	AUC	pAUC	TPR	FPR	TNR	FNR	FDR	MER	AUC	pAUC
	**Without outlying expressions**								**For 1 outlier with each of 5% genes**							
ANOVA	0.928	0.001	0.999	0.072	0.072	0.003	0.916	0.183	0.472	0.011	0.989	0.527	0.527	0.021	0.473	0.094
SAM	0.940	0.001	0.999	0.060	0.060	0.002	0.939	0.188	0.477	0.011	0.989	0.522	0.522	0.021	0.476	0.095
LIMMA	0.945	0.001	0.999	0.055	0.055	0.002	0.944	0.189	0.475	0.011	0.989	0.525	0.525	0.021	0.477	0.095
eLNN	0.927	0.001	0.999	0.072	0.072	0.003	0.926	0.185	0.425	0.012	0.988	0.575	0.575	0.023	0.424	0.085
EBarrays	0.932	0.001	0.999	0.068	0.068	0.003	0.933	0.186	0.398	0.012	0.988	0.603	0.603	0.024	0.399	0.080
BetaEB	0.932	0.001	0.999	0.068	0.068	0.003	0.931	0.186	0.930	0.001	0.999	0.070	0.070	0.003	0.931	0.186
KW	0.948	0.001	0.999	0.052	0.052	0.002	0.949	0.190	0.477	0.011	0.989	0.522	0.522	0.021	0.476	0.096
Proposed	0.928	0.001	0.999	0.072	0.072	0.003	0.918	0.183	0.925	0.002	0.998	0.075	0.075	0.003	0.917	0.183
	**For 1 outlier with each of 10% genes**								**For 1 outlier with each of 75% genes**							
ANOVA	0.320	0.014	0.986	0.680	0.680	0.027	0.321	0.064	0.087	0.019	0.981	0.912	0.912	0.036	0.087	0.018
SAM	0.325	0.014	0.986	0.675	0.675	0.027	0.324	0.065	0.087	0.019	0.981	0.912	0.912	0.036	0.086	0.018
LIMMA	0.323	0.014	0.986	0.677	0.677	0.027	0.323	0.064	0.092	0.019	0.981	0.907	0.907	0.036	0.091	0.018
eLNN	0.258	0.015	0.985	0.743	0.743	0.030	0.256	0.051	0.040	0.020	0.980	0.960	0.960	0.038	0.041	0.008
EBarrays	0.230	0.016	0.984	0.770	0.770	0.031	0.231	0.046	0.032	0.020	0.980	0.968	0.968	0.039	0.033	0.006
BetaEB	0.930	0.001	0.999	0.070	0.070	0.003	0.931	0.186	0.032	0.020	0.980	0.968	0.968	0.039	0.031	0.006
KW	0.323	0.014	0.986	0.677	0.677	0.027	0.324	0.064	0.087	0.019	0.981	0.912	0.912	0.036	0.086	0.018
Proposed	0.924	0.002	0.998	0.076	0.076	0.003	0.916	0.183	0.907	0.002	0.998	0.092	0.092	0.004	0.908	0.181

Average performance results of eight methods (ANOVA, SAM, LIMMA, eLNN, EBarrays, BetaEB, KW and Proposed) based on 100 datasets generated using a one-way ANOVA model with m=2 groups/conditions and $\sigma^2 = 0.05$ for sample size n1=n2=4. Each dataset for each case contained 300 true DE genes, and the remainder were 19700 true EE genes. The performance indices/measures TPR, FPR, TNR, FNR, FDR, MER and AUC were calculated for each method based on the top 300 estimated DE genes, under the assumption that the other estimated genes in each dataset for each case were EE genes for each method. The performance measure pAUC was calculated at FPR=0.2 for each method and for each dataset.

Table B: Performance evaluation based on simulated gene expression profiles using a Bayesisn model (EBarrays LNN-model) with m=2 conditions

Results for the small-sample case ($\mathbf{n_1 = n_2 = 3}$)																
Methods	TPR	FPR	TNR	FNR	FDR	MER	AUC	pAUC	TPR	FPR	TNR	FNR	FDR	MER	AUC	pAUC
	**Without outlying expressions**								**For 1 outlier with each of 5% genes**							
ANOVA	0.818	0.005	0.995	0.182	0.182	0.010	0.803	0.159	0.420	0.012	0.988	0.580	0.580	0.023	0.419	0.084
SAM	0.760	0.005	0.995	0.240	0.240	0.010	0.761	0.152	0.420	0.012	0.988	0.580	0.580	0.023	0.422	0.084
LIMMA	0.797	0.004	0.996	0.203	0.203	0.008	0.796	0.159	0.425	0.012	0.988	0.575	0.575	0.023	0.424	0.085
eLNN	0.820	0.004	0.996	0.180	0.180	0.007	0.822	0.164	0.453	0.011	0.989	0.547	0.547	0.022	0.453	0.089
EBarrays	0.823	0.004	0.996	0.177	0.177	0.007	0.824	0.164	0.388	0.012	0.988	0.613	0.613	0.024	0.386	0.077
BetaEB	0.823	0.004	0.996	0.177	0.177	0.007	0.822	0.164	0.823	0.004	0.996	0.177	0.177	0.007	0.823	0.164
KW	0.850	0.003	0.997	0.150	0.150	0.004	0.795	0.181	0.460	0.011	0.989	0.540	0.540	0.022	0.461	0.092
Proposed	0.818	0.005	0.995	0.182	0.182	0.010	0.803	0.159	0.815	0.009	0.991	0.185	0.185	0.009	0.802	0.158
	**For 1 outlier with each of 10% genes**								**For 1 outlier with each of 75% genes**							
ANOVA	0.282	0.015	0.985	0.718	0.715	0.029	0.283	0.056	0.077	0.019	0.981	0.922	0.922	0.037	0.078	0.015
SAM	0.282	0.015	0.985	0.718	0.718	0.029	0.284	0.056	0.082	0.019	0.981	0.917	0.917	0.037	0.083	0.016
LIMMA	0.285	0.015	0.985	0.715	0.715	0.029	0.283	0.057	0.082	0.019	0.981	0.917	0.917	0.037	0.081	0.016
eLNN	0.338	0.014	0.986	0.662	0.662	0.026	0.337	0.066	0.075	0.019	0.981	0.925	0.925	0.037	0.076	0.015
EBarrays	0.235	0.016	0.984	0.765	0.765	0.031	0.238	0.047	0.035	0.020	0.980	0.965	0.965	0.039	0.036	0.007
BetaEB	0.818	0.004	0.996	0.182	0.182	0.007	0.818	0.163	0.035	0.020	0.980	0.965	0.965	0.039	0.033	0.007
KW	0.305	0.014	0.986	0.695	0.695	0.028	0.305	0.062	0.077	0.019	0.981	0.922	0.922	0.037	0.077	0.015
Proposed	0.816	0.010	0.990	0.184	0.184	0.009	0.805	0.158	0.813	0.10	0.990	0.187	0.187	0.011	0.727	0.154

Results for the large-sample case ($\mathbf{n_1 = n_2 = 15}$)																
Methods	TPR	FPR	TNR	FNR	FDR	MER	AUC	pAUC	TPR	FPR	TNR	FNR	FDR	MER	AUC	pAUC
	**Without outlying expressions**								**For 1 or 2 outliers with each of 5% genes**							
ANOVA	0.941	0.001	0.999	0.059	0.059	0.002	0.942	0.187	0.537	0.009	0.991	0.463	0.463	0.018	0.536	0.107
SAM	0.942	0.001	0.999	0.058	0.058	0.002	0.941	0.188	0.537	0.009	0.991	0.463	0.463	0.018	0.535	0.107
LIMMA	0.942	0.001	0.999	0.058	0.058	0.002	0.944	0.188	0.540	0.009	0.991	0.460	0.460	0.018	0.541	0.108
eLNN	0.938	0.001	0.999	0.062	0.062	0.002	0.939	0.185	0.463	0.011	0.989	0.537	0.537	0.021	0.463	0.092
EBarrays	0.942	0.001	0.999	0.058	0.058	0.002	0.943	0.188	0.590	0.008	0.992	0.410	0.410	0.016	0.591	0.118
BetaEB	0.942	0.001	0.999	0.058	0.058	0.002	0.944	0.188	0.942	0.001	0.999	0.058	0.058	0.002	0.942	0.188
KW	0.940	0.001	0.999	0.060	0.060	0.002	0.941	0.186	0.820	0.004	0.996	0.180	0.180	0.007	0.821	0.164
Proposed	0.941	0.001	0.999	0.059	0.059	0.002	0.942	0.187	0.935	0.001	0.999	0.065	0.065	0.003	0.936	0.187
	**For 1 or 2 outliers with each of 10% genes**								**For 1 or 2 outliers with each of 75% genes**							
ANOVA	0.398	0.012	0.988	0.603	0.603	0.024	0.397	0.079	0.285	0.015	0.985	0.715	0.715	0.029	0.284	0.056
SAM	0.400	0.012	0.988	0.600	0.600	0.024	0.400	0.080	0.285	0.015	0.985	0.715	0.715	0.029	0.284	0.056
LIMMA	0.400	0.012	0.988	0.600	0.600	0.024	0.400	0.080	0.310	0.014	0.986	0.690	0.690	0.028	0.309	0.061
eLNN	0.412	0.012	0.988	0.588	0.588	0.024	0.412	0.082	0.407	0.012	0.988	0.593	0.593	0.024	0.407	0.082
EBarrays	0.445	0.011	0.989	0.555	0.555	0.022	0.445	0.089	0.265	0.015	0.985	0.735	0.735	0.029	0.265	0.053
BetaEB	0.940	0.001	0.999	0.060	0.060	0.002	0.940	0.188	0.265	0.015	0.985	0.735	0.735	0.029	0.265	0.053
KW	0.828	0.004	0.996	0.172	0.172	0.007	0.827	0.165	0.907	0.002	0.998	0.092	0.092	0.004	0.907	0.181
Proposed	0.932	0.001	0.999	0.068	0.068	0.003	0.935	0.186	0.930	0.001	0.999	0.070	0.070	0.003	0.930	0.184

Average performance results for comparison of eight methods ANOVA, SAM, LIMMA, eLNN, EBarrays, BetaEB, KW and Proposed) based on 100 datasets generated using a Bayesisn model (EBarrays LNN-model) with m=2 groups/conditions for both small- and large-sample cases (n1=n2 = 3 and 15). Each dataset for each case contained 300 true DE genes, and the remainder were 19700 true EE genes. The performance measures/indices (PM/PI) TPR, FPR, TNR, FNR, FDR, MER and FDR were calculated for each method based on the top 300 estimated DE genes, under the assumption that the other estimated genes in each dataset for each case were EE genes for each method. The performance measure pAUC was calculated at FPR=0.2 for each method and for each dataset.

2.2. Supplementary Results of Colon Data Analysis

Table C: Up/down-regulated in Colon Cancer According to Oncomine Database

Study	Description	Fold Change	P	Number of Measured Genes	Overexpression/ Under-expression Gene Rank	References
		**MUC2 is down-regulated in colon cancer according to oncomine database**				
1	Colorectal Carcinoma (41) vs. Normal (5)	-3.797	8.10E-5	19,574	1701 (in top 9%)	Genome Biol 2007/07/05
2	Colon Adenocarcinoma (70) vs Normal (21)	-6.478	3.73E-12	19,574	596 (in top 4%)	Clin Exp Metastasis 2010/02/01
4	Colon Carcinoma (5) vs Normal (10)	-17.908	5.00E-5	19,574	1822 (in top 10%)	PLoS One 2010/10/01
		**UBE2I is upregulated in colon cancer according to oncomine database**				
1	Colon Adenocarcinoma (18) vs. Normal (18)	1.543	9.02E-5	4,321	136 (in top 4%)	Cancer Res 2001/04/01
2	Colon Adenocarcinoma (39) vs. Normal (22)	1.479	9.34E-4	1,527	100 (in top 7%)	Proc Natl Acad Sci U S A 1999/06/08
		**PRIM1 is upregulated in colon cancer according to oncomine database**				
1	Colon Carcinoma (5) vs. Normal (10)	2.827	2.39E-4	19,574	4009 (in top 21%)	PLoS One 2010/10/01
2	Colon Adenocarcinoma (39) vs Normal (22)	1.418	2.58E-4	1,527	54 (in top 4%)	Proc Natl Acad Sci U S A 1999/06/08
3	Colon Adenocarcinoma (102) vs Normal (19)	1.894	2.14E-15	20,423	1027 (in top 6%)	Nature 2012/07/18
4	Colon Adenocarcinoma (50) vs Normal (28)	1.426	1.78E-6	9,256	1103 (in top 12%)	Int J Cancer 2007/11/01
		**ADCY2 is down-regulated in colon cancer according to oncomine database**				
1	Colon Adenocarcinoma (50) vs Normal (28)	-1.262	1.36E-7	9,256	399 (in top 5%)	Int J Cancer 2007/11/01
		**POLD2 is Up-regulated in colon cancer according to oncomine database**				
1	Colon Adenocarcinoma (18) vs Normal (18)	2.172	0.001	4,321	278 (in top 7%)	Cancer Res 2001/04/01

Continued on next page

Table C — *Continued from previous page*

Study	Description	Fold Change	P	Number of Measured Genes	Overexpression/ Under-expression Gene Rank	References
2	Colon Carcinoma (5) vs. Normal (10)	2.737	7.30E-9	19,574	550 (in top 3%)	PLoS One 2010/10/01
		**REG1A is upregulated in colon cancer according to oncomine database**				
1	Colon Adenocarcinoma (50) vs. Normal (28)	9,256	1.05E-16	4,321	85 (in top 1%)	Int J Cancer 2007/11/01
2	Colon Carcinoma (5) vs. Normal (10)	2.523	0.067	19,574	8059 (in top 42%)	PLoS One 2010/10/01
		**GLUT4 is down-regulated in colon cancer according to oncomine database**				
1	Colon Adenocarcinoma (284) vs. Normal (90)	-1.260	1.23E-51	18,823	269 (in top 2%)	Nature 2012/07/18
2	Colon Adenocarcinoma (39) vs. Normal (22)	-1.680	0.002	1,527	101 (in top 7%)	PLoS One 2010/10/01

***Available at: http://www.oncomine.org.**

References

[1] Mood AM, Graybill FA and Boes DC. Introduction to the theory of statistics (3rd edition, 1974). McGraw Hill, New York, NY.

[2] Mollah MNH, Minami M, and Eguchi S: Robust prewhitening for ICA by minimizing β-divergence and its application to FastICA. *Neural Processing Letters 2007*, **25**(2), 91-110.

[3] Mollah MNH., Sultana N, Minami M. and Eguchi S: (Robust Extraction of Local Structures by the Minimum β-Divergence method. *Neural Network 2010*, **23**, 226-238.
